# Insights from systems pharmacology into cardiovascular drug discovery and therapy

**DOI:** 10.1186/s12918-014-0141-z

**Published:** 2014-12-24

**Authors:** Peng Li, Yingxue Fu, Jinlong Ru, Chao Huang, Jiangfeng Du, Chunli Zheng, Xuetong Chen, Pidong Li, Aiping Lu, Ling Yang, Yonghua Wang

**Affiliations:** Center of Bioinformatics, College of Life Science, Northwest A and F University, Yang ling, Shaanxi 712100 China; Department of Biochemistry, Cardiovascular Research Institute Maastricht, Maastricht University, Maastricht, the Netherlands; School of Chinese Medicine, Hong Kong Baptist University, Kowloon Tong, Hong Kong; Lab of Pharmaceutical Resource Discovery, Dalian Institute of Chemical Physics, Chinese Academy of Sciences, Dalian, Liaoning China

**Keywords:** Cardiovascular disease, Network pharmacology, Network analysis, Drug discovery, Drug-target network, Gene-disease network

## Abstract

**Background:**

Given the complex nature of cardiovascular disease (CVD), information derived from a systems-level will allow us to fully interrogate features of CVD to better understand disease pathogenesis and to identify new drug targets.

**Results:**

Here, we describe a systematic assessment of the multi-layer interactions underlying cardiovascular drugs, targets, genes and disorders to reveal comprehensive insights into cardiovascular systems biology and pharmacology. We have identified 206 effect-mediating drug targets, which are modulated by 254 unique drugs, of which, 43% display activities across different protein families (sequence similarity < 30%), highlighting the fact that multitarget therapy is suitable for CVD. Although there is little overlap between cardiovascular protein targets and disease genes, the two groups have similar pleiotropy and intimate relationships in the human disease gene-gene and cellular networks, supporting their similar characteristics in disease development and response to therapy. We also characterize the relationships between different cardiovascular disorders, which reveal that they share more etiological commonalities with each other rooted in the global disease-disease networks. Furthermore, the disease modular analysis demonstrates apparent molecular connection between 227 cardiovascular disease pairs.

**Conclusions:**

All these provide important consensus as to the cause, prevention, and treatment of various CVD disorders from systems-level perspective.

**Electronic supplementary material:**

The online version of this article (doi:10.1186/s12918-014-0141-z) contains supplementary material, which is available to authorized users.

## Background

In recent years, the prospect for the cardiovascular disease (CVD) pharmacotherapy seems to have ‘hit the wall’, with multiple high-profile trial failures and declining industrial interest. Reasons for such predicament might include an intensive regulatory environment, a competitive market, the elevated bar of existing medicines for further innovation and the increasing cost of mega-trials. However, the most important and intrinsic reason comes up to the lack of mechanistic understanding of drug action and the complicated etiologies [1-4].

Over the past 50 years, sorts of blockbusters for the therapy of CVD have been sprung up, such as statins, angiotensin-converting enzyme (ACE) inhibitors, anti-platelet agents and beta-blockers. However, many of these drugs play functional roles in biological processes outside the scope of the drug’s intended effects [5,6]. This often leads to unexpected situations at various stages during the drug discovery process. For example, torcetrapib (Pfizer, New York, NY, USA), an inhibitor of cholesteryl ester transfer protein (CETP), failed in the Investigation of Lipid Level Management to Understand Its Impact in Atherosclerotic Events (ILLUMINATE) trial for the increased risk of mortality and morbidity [7], due to the off-target effects of torcetrapib on hypertension [8]. On the contrary, the unpredictable off-target interaction may also give rise to safety effects on patients. For example, statins, originally designed to target elevated lipids for the treatment of atherosclerosis, might also confer cardiovascular benefit with their anti-inflammatory effects, independent of LDL-lowering effects [9].

Indeed, a growing body of post-genomic biology (as reflected for acquisition of high-throughput genomic, transcriptomic, proteomic, and metabolomic data) has been revealing a far more complex portrait of drug actions. It is appreciated that many drugs with a specific efficacy actually act on multiple protein targets [10,11]. This so-called polypharmacology is an undesirable property in the conventional reductionist paradigm and might be more suitable to view through the lens of systems-based approaches [11].

The complexity of CVD also resists traditional efforts which have been applied to identify a single gene or pathway to treat the disease [3,12]. Common forms of CVD are caused by multiple genetic factors, each of which contributes modestly to the disease risk, and also environmental factors. Genetically, it has become evident that many human diseases cannot be attributed to the malfunction of a single gene but to complex interactions among multiple genetic variants. Perturbations in several genes might only make subtle contributions to the susceptibility of a particular individual [3]. Therefore, the disease causations should be studied on the basis of the entire body of knowledge including all genes that are associated with the clinical traits. Epidemiologically, cardiovascular events are not only related to environmental factors such as smoking, diet and physical activity but also linked to other systemic disorders such as hypertension, diabetes, obesity, or thyroid disease [4]. Traditional research efforts normally address these individual risk factors in isolation, even though they are believed to concomitantly contribute to the disease pathogenesis (disease comorbidity) [13]. Accordingly, a systems-based approach integrating all the potential related factors involved in the pathologies and disease treatment is required to address these complex issues.

**Figure 1:**
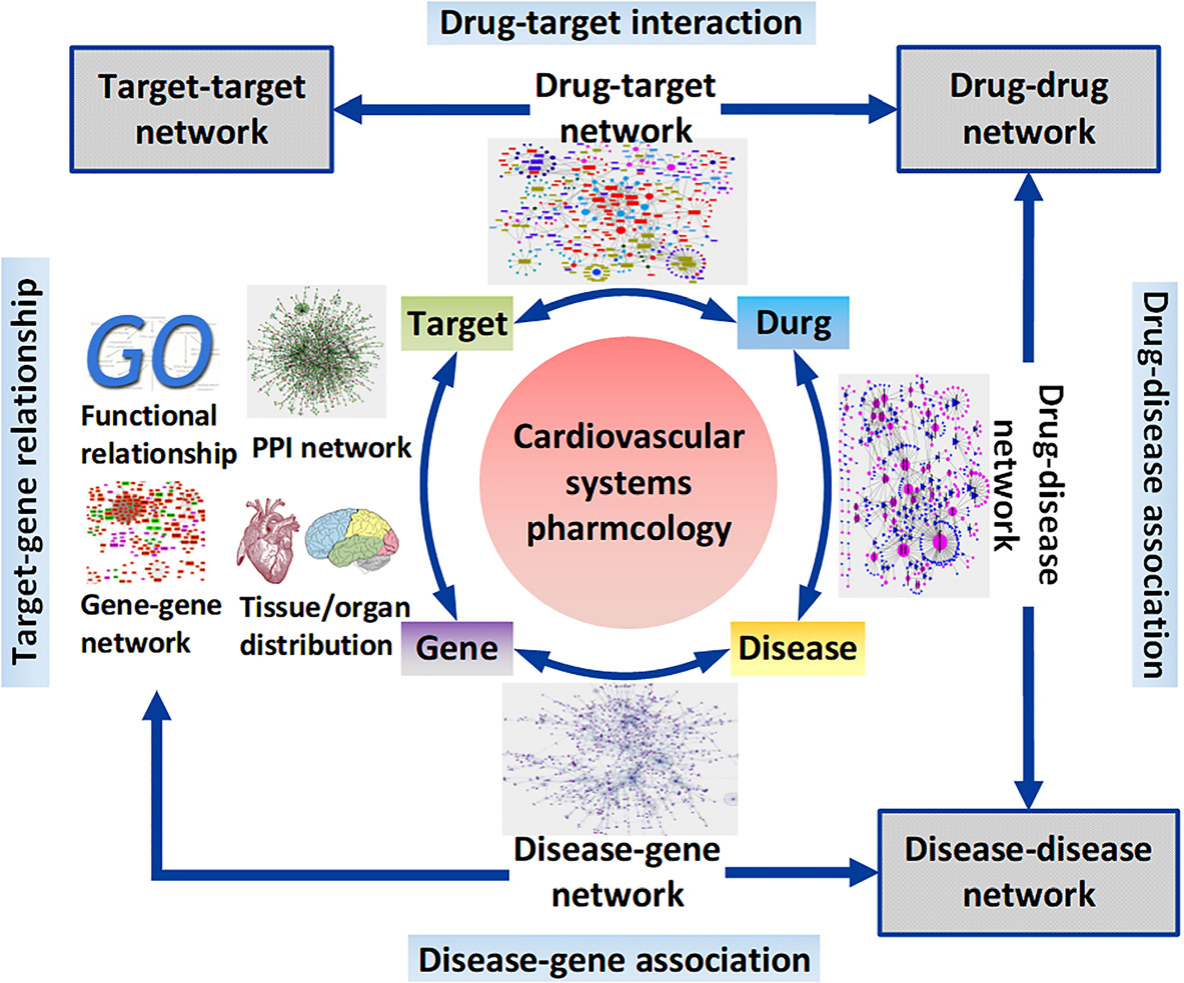
**Network analysis serves an integral role in cardiovascular systems pharmacology.** The drug–target network is built by connecting the cardiovascular drugs with their corresponding cardiovascular targets. Starting from this graph, it generates two biologically relevant network projections: the target–target network and the drug-drug network. In the target–target network, nodes represent targets, and two protein targets are connected to each other if they share at least one drug. In the drug–drug network, nodes represent drugs, and two drugs are connected if they are associated with the same protein target. A bipartite graph of gene-disease associations is constructed in which a gene and a disorder are connected if mutations in that gene are implicated in that disorder. From the gene-disease network, two biologically relevant network projections were generated. In the human disease gene-gene network, every two genes are applied to connect with a common disease based on the global gene-disease associations. The gene disease-disease network is transformed by connecting two disorders if they are associated with the same gene based on the gene-disorder associations. The drug-disease network is constructed by mapped the approved cardiovascular drugs to their corresponding indications. Physical interactions between proteins can also be used to produce the human protein-protein interaction (PPI) network. For cardiovascular pharmacology, these interaction networks will provide a global template for computational and mathematical systems modeling, simulation, and prediction.

To quantitatively characterize the complex relationships between cardiovascular drugs, targets, disease genes and disorders, we construct a series of networked relationships including cardiovascular drug-target, gene-disease, drug-disease, and protein-protein interaction networks by integrating publicly available drug data (See Figure 1 for an overview of the analysis process). We believe that within- and between-studies of these networks will provide a more comprehensive and profound understanding of the cardiovascular disease pathogenesis and drug action. Herein, we apply integrated network analysis and mainly focus on three areas that are critical to cardiovascular systems biology and pharmacology: 1) the extent of polypharmacological effects of cardiovascular agents, 2) the relationships between drug targets and disease genes in biological networks, and 3) the genetic and molecular connections between different cardiovascular diseases. In addition, all these CVD-associated factors and their multi-layer interactions are integrated and provided in a comprehensive database CVDSP for readers to explore information interactively (http://sm.nwsuaf.edu.cn/lsp/cvdsp.php).

## Methods

### Compiling cardiovascular drug and their therapeutic targets

The complete cardiovascular drug-target information was downloaded from the DrugBank database [14], therapeutic target database (TTD) [15] and FDA orange book [16] as of November 2012. The resulting list of drug targets was manually inspected one by one by literature curation to assure the quality of the data. We classified drugs and target proteins according to therapeutic areas and functional family, respectively. The reproducible set of interactions, pharmacological activities of drugs and function annotations of targets were provided in supplementary information as a resource for researchers who are interested in the cardiovascular pharmacology (Additional file 1). The curation of the drug-target data set involved the identification of 254 approved cardiovascular drugs with 206 successful cardiovascular protein targets. This data set was used to build the drug-target network.

### Compiling genetic phenotypes and phenotype-associated genes

The most complete and best-curated list of known phenotype-gene associations is maintained in the Morbid Map (MM) of the Online Mendelian Inheritance in Man (OMIM) [17]. Each entry of the MM is composed of four fields, the name of the disorder, the associated gene symbols, its corresponding OMIM id, and the chromosomal location. We analyzed the complete data set and performed a manual curation following procedure of the visionary study by Goh *et al.* [18]. We downloaded the MM file on January 2013. Out of 6,252 MM entries, we selected 4,811 entries with the “(3)” tag, for which there is strong evidence that at least one mutation in the particular gene is causative to the phenotype. We then parsed these 4,811 phenotype terms into 1,775 distinct phenotypes by merging phenotype subtypes of a single phenotype, based on their given names and corresponding Medical Subject Headings (MeSH) [19] vocabulary on January, 2013. The merging was done first automatically and then each entry was verified manually. Each disease was then assigned a unique disease ID.

The curated data set contained 1,775 phenotypes and 3,039 associated genes (Additional file 1), of which 98 are cardiovascular disorders associated with 268 genes (Additional file 1). In addition, 111 disease genes encode the cardiovascular target proteins, of which 35 overlaps the cardiovascular genes associated with 26 cardiovascular disorders (Additional file 1).

We constructed disease gene-gene network (DGG network; Additional file 1) and gene disease-disease network (GDD network; Additional file 1) which were derivative from the gene-disease associations (Additional file 1). In the GG network, every two genes are applied to connect with a common disease based on the global gene-disease associations. Similarly, the GDD network is transformed by connecting two disorders if they are associated with the same gene in the gene-disorder associations.

### Generating a disease modular network

A network was generated by determining the first-order interactions of cardiovascular gene products associated with a given phenotypic subgroup in the PPI network. Interactions of the cardiovascular gene products were integrated into a network by always including direct interactions between cardiovascular gene products, and only including interactions with other proteins above a network score threshold. The network score for a protein is the amount of interactions to cardiovascular gene products out of all interaction partners of the protein, making networks consisting of proteins with many interactions less important and reducing noise from highly interacting proteins for non-cardiovascular proteins. The median of all scores for all non-cardiovascular proteins is 0.25 and is used as the threshold-score [20]. Detailed views of the networks can be seen in Additional file 1.

### Compiling a high-quality, comprehensive list of binary protein-protein interactions

Human protein-protein interaction (PPI) set were assembled from HINT (High-quality protein interactomes) [21] updated June 3, 2013. HINT is a database of high-quality PPIs integrated from various sources and filtered to remove low-quality/erroneous interactions. The resulting set of PPIs contained 28,629 non-self-interacting, non-redundant interactions between 8,495 proteins, of which 132 were cardiovascular targets and 191 were cardiovascular gene products mapped by Gene names. The list of PPIs used is available at the online database CVDSP (http://sm.nwsuaf.edu.cn/lsp/cvdsp.php).

### Assessing molecular connections between disorders

To quantify the cellular network-level relationship between pair of phenotypes, we assessed the molecular associations for each pair of phenotype modules by their shared protein-protein interactions in the disease modular network. Number of shared protein-protein interactions is the number of protein-protein interactions that link genes between the two modules. The significance of shared protein-protein interactions was measured by randomization tests of the resulting network. For two phenotype modules, we firstly randomly generated two modules with the same number of disease genes. We then calculated the numbers of shared protein-protein interactions between the two random modules. This procedure was performed for 10, 000 times to obtain significant statistics and *P* values for the two disorders. All pairs of disorders involving shared protein-protein interactions and *P* values are listed in Additional file 1.

### Topological features of a network

The degree of a node is the number of edges connecting to the node. The shortest path between two nodes is the path with the smallest number of links between the selected nodes. The betweenness (centrality) denotes the proportion of all shortest paths between node pairs in a network passing through the measured node, indicating the relative importance of the particular node in network global connectivity. Closeness (centrality) is defined as the inverse sum of shortest distances to all other nodes from a focal node, indicating the expected time from a focal node to reach others. The clustering coefficient is defined as *C*_*i*_ = 2*n*/*k*_*i*_ (*k*_*i*_ – 1), where *n* is the number of direct links connecting the *k*_*i*_ nearest neighbors of node *i*. The average of *C*_*i*_ over all nodes of a network assesses network modularity.

### Calculating the functional similarity between cardiovascular targets and genes

To validate the intimate relationship between cardiovascular targets and genes derived from the network properties, we calculated the GO-based semantic similarity between cardiovascular targets and genes. We firstly downloaded Biological Process (BP), Cellular Component (CC), or Molecular Function (MF) branches of the Gene Ontology (GO) from the GO database [22]. GO-based semantic similarity scores (GSS) between cardiovascular targets and genes were calculated according to Resnik [23], using the csbl.go R package [24] selecting the option to use all three ontologies. We calculated the average GSS of all pairs of cardiovascular target and gene. Random controls were obtained by selecting the same number of genes 10, 000 times randomly to control for cardiovascular genes. All statistics are shown in Additional file 1.

### Database development

To accompany the findings from this study, an online database CVDSP (http://sm.nwsuaf.edu.cn/lsp/ cvdsp.php) was developed to allow researchers to access the underlying information in a user-friendly manner. We have included all of our data sets in this database. The drug-target interactions, gen-phenotype associations, drug-indication associations and target-gene relationships as well as their derivate networks such as drug-drug and gene-gene networks can be explored interactively. We will regularly update our data sets and the website to keep up with the growth of the databases used.

### Statistical Analysis

All the t-tests and z-tests were done in Mathematica (Wolfram Research) using the HypothesisTests package. Kolmogorov-Smirnov and Wilcoxon rank sum tests were done in Matlab (Mathworks) using the “kstest2” and “ranksum” commands, respectively. All the error terms in the text and the figures are the standard errors.

## Results

### Classification of cardiovascular drugs and their therapeutic targets

The careful curation of the drug-target data set involves the identification of approved cardiovascular drugs with successful cardiovascular protein targets. This results in a list of 254 drugs that act on 206 protein targets (Additional file 1). Eleven drug classes are identified according to the Anatomical Therapeutic Chemical (ATC) rule. The biggest ATC class in the data set is for cardiac therapy (49 drugs), followed by antithrombotic agents (42 drugs), antihypertensives (34 drugs), agents acting on the renin-angiotensin system (23 drugs), diuretics (23 drugs), beta blockers (22 drugs), lipid modifying agents (18 drugs), vasoprotectives (15 drugs), calcium channel blockers (15 drugs), peripheral vasodilators (7 drugs) and etc. (Table 1). The drug-target association data show that 59 receptors are the main targets for the cardiovascular agents, weighing ~28.6% of all cardiovascular targets (Table 2). G protein-coupled receptors (GPCRs) are the most common class of the receptor targets, comprising ~72.9% of all cardiovascular receptors. ~34.6% of the drugs target GPCRs and are mainly involved in cardiac and anti-hypertension therapies. This is consistent with the central role of GPCRs in cardiovascular biology [25]. The other common receptors are nuclear receptors, comprising ~15.2% of all cardiovascular receptor targets, and these receptors are mainly targeted by vasoprotectives.

**Table 1:** **Classification of cardiovascular drugs**

**Drug class**	**Numbers**
B01 Antithrombotic agents	42
C01 Cardiac therapy	50
C02 Antihypertensives	34
C03 Diuretics	23
C04 Peripheral vasodilators	7
C05 Vasoprotectives	15
C07 Beta blocking agents	22
C08 Calcium channel blockers	15
C09 Agents acting on the renin-angiotensin system	23
C10 Lipid modifying agents	18
X Dual function	7

**Table 2:** **Classification of cardiovascular targets**

**Target class**	**Numbers**	**Target child class**	**Numbers**
Receptor	68	GPCR	52
		Nuclear Receptor	9
		Other Receptor	7
Transporter	65	Ion Channel	53
		Solute Carrier	8
		Other Transporter	4
Enzyme	55	EC1	12
		EC2	6
		EC3	30
		EC4	6
		EC5	1
Other	27	Cytokine	15
		Integrins	3
		Annexin	2
		Calmodulin	2
		Antifibrotic Factor	1
		Calnexin	1
		Calreticulin	1
		Fibrinogen	1
		Transcription Factor	1

Transporters make up the second largest group of drug targets: 65 proteins (~31.6% of all cardiovascular targets) are transporters, and ~81.5% of them are ion channels targeted by 63 drugs (~25% of all approved cardiovascular drugs). Ion channels have been commonly targeted by calcium channel blockers. Solute carriers, the second largest transporter target class, are mostly targeted by diuretics.

Enzymes are the third key class of cardiovascular targets, with 55 proteins in the class, comprising ~26.7% of all cardiovascular targets. Among the enzyme target list, Hydrolases (EC 3) are dominant, comprising more than half of all cardiovascular enzyme targets. They are followed by oxidoreductases (EC 1), which comprises ~22% of all cardiovascular enzyme targets. Antithrombotic agents and the agents acting on the renin-angiotensin system (RAS) normally hit the enzyme targets. Other common enzyme targets include 6 transferases (EC 2), 6 lyases (EC 4) and 1 isomerase (EC 5).

### Quantify the polypharmacology for cardiovascular drugs and targets

**Figure 2:**
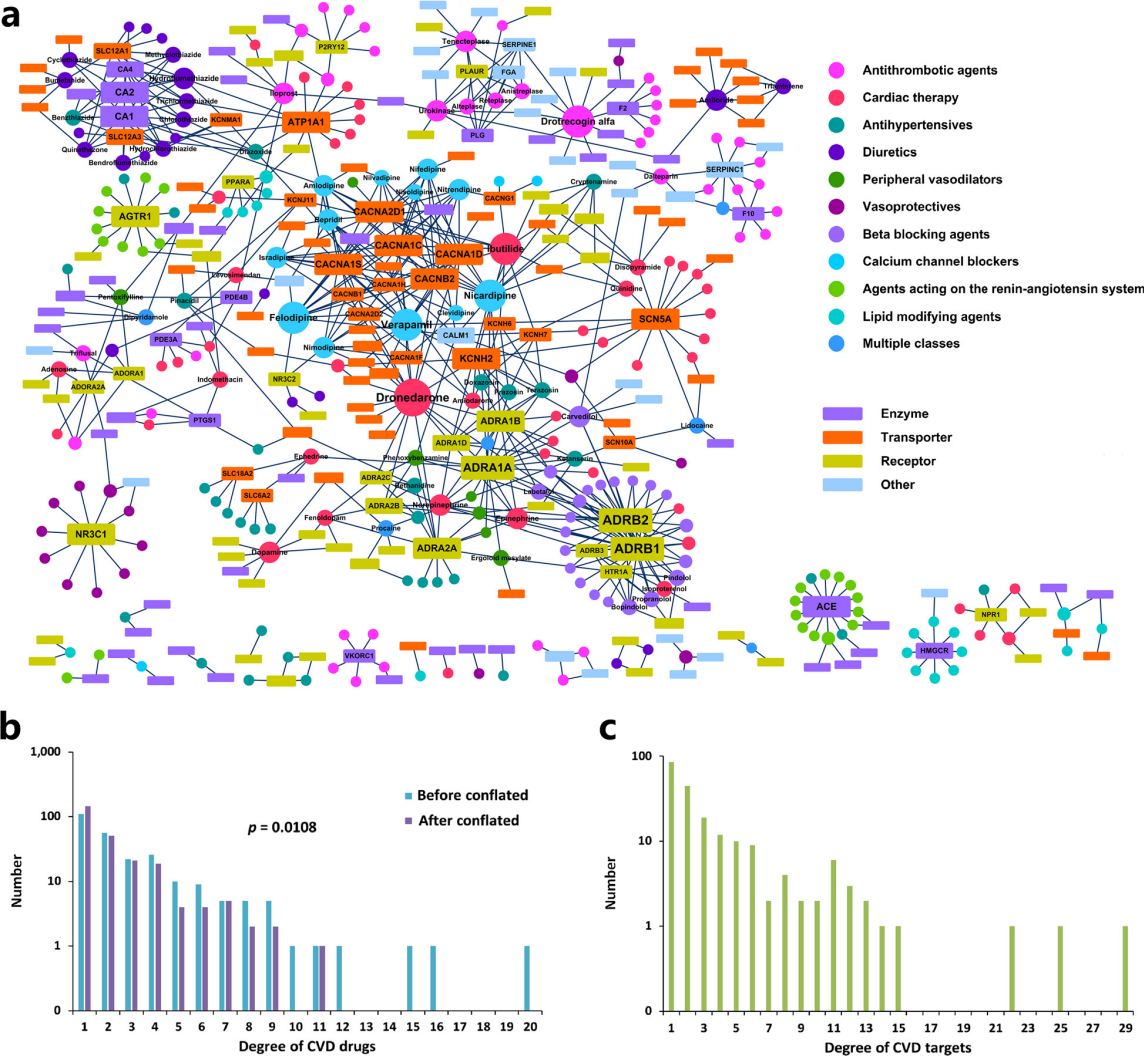
**The drug–target network. (a)** The drug-target network was generated from the known associations between FDA-approved cardiovascular drugs and their target proteins. Nodes represent drugs (shown as circles) and targets (shown as rectangles). A link is placed between a drug and a target node if the protein is a known target of the drug. The size of the drug (protein) node is proportional to the number of the relevant targets (the number of the relevant drugs). Drugs are colored according to their Anatomical Therapeutic Chemical (ATC) Classification, and targets are colored according to protein family obtained from the Uniprot database. **(b)** Distribution of target proteins for drugs (drug node degrees) in the drug-target network. This distribution shows most cardiovascular drugs target a small number of targets, but some of them have many targets. **(c)** Distribution of drugs for their targets (target node degrees) in the drug-target network. Most targets have a few drugs, but some targets have many drugs.

Massive studies have revealed that the drug promiscuity is a phenomenon much more common than previously thought and is critically important for drug discovery, especially for the complex diseases such as CVD, which is usually multiple genes involved diseases [26-28]. For example, amiodarone exerts its antiarrhythmic effect by acting on adrenergic receptors and potassium and calcium channels, simultaneously (Additional file 1). However, there is still lack of quantification of the degree of polypharmacological effects of cardiovascular drugs. The drug-target network offers a panoramic view for the drug-target interaction landscape, permitting to explore comprehensive information on cardiovascular pharmacology from molecules to systems, including the overall degree of polypharmacological effects of cardiovascular agents on various targets [6]. Here, the cardiovascular drug–target network was built by connecting the 254 approved cardiovascular drugs with their corresponding 206 cardiovascular targets (Figure 2a). The overall network contains 701 drug-target connections, in which 198 drugs (~78% of the total) and 165 targets (~80% of the total) compose the largest connected component of the network (Figure 2a), reflecting high interconnectedness between the drugs and their targets. To quantify the polypharmacological effect, we counted the number of cardiovascular targets for each drug, that is, the degree for each drug node in the drug-target network (See Methods). The degree distribution of drug nodes indicates that most drugs acting on more than one target, and the average number of target proteins per drug is 2.8. Interestingly, some drugs even have dozens of targets, such as Verapamil (16 targets) and dronedarone (20 targets) (Figure 2b). These properties suggest the promiscuity of cardiovascular drugs.

However, the above measurement neglects the fact that some drugs target homologous proteins, which can inflate the polypharmacological effects of these drugs. As we know, drugs that target homologous proteins should be less promiscuous than those have demonstrable activity across different protein families [29]. For example, the activity of cryptenamine, an antihypertensive drug, mainly depends on muscarinic acetylcholine receptors, including CHRM1, CHRM2, CHRM3, CHRM4 and CHRM5 (Additional file 1). In contrast, dronedarone, an antiarrhythmic agent, acts on multiple targets including sodium, potassium and calcium channels and various adrenergic receptors for management of atrial fibrillation (Additional file 1). Obviously, the degree of polypharmacology for cyptenamine is correspondingly weaker than that of dronedarone. Indeed, of all 145 promiscuous drugs in our dataset, more than 65% have been found to display activities against some proteins from the similar family (sequence similarity ≥ 30%). This is also reflected by the target-target network, in which nodes represent targets, and two protein targets are connected to each other if they share at least one drug (Additional file 1). In this network, 200 out of 206 targets are connected to other proteins. Drugs with multiple targets are responsible for this high interconnectedness. It is evident that some specific target classes, such as voltage-dependent calcium channel protein family, tend to cluster together with common drugs (calcium channel blockers). Moreover, it is also found that these targets mostly belong to the same functional family.

To eliminate this bias effect caused by homologous proteins in the polypharmacology analysis, we have conflated the homology target proteins into their specific families according to rule of sequence similarity ≥ 30% to build a modified drug-target network. After removing the paralogous genes, the degree distributions of the drugs between the modified and original drug-target network only show a slight variation (*p* = 0.0108, Two-sample Kolmogorov-Smirnov test; Figure 2b). For example, the number of drugs with one target increases from 109 to 145. However, although the degrees of some drugs are reduced, we still observe a significant proportion (43%) of drugs target more than one protein, indicating the promiscuous nature of cardiovascular drugs is not significantly changed. Such polypharmacological information enables a rational approach to selecting multiple candidates for CVD treatment. It should be noted that, most of these known promiscuous drugs are discovered based on the traditional phenotypic-screening assays [10,30], which normally did not distinguish the explicit therapeutic targets and their underlying molecular interactions [31]. Therefore, follow-up studies concerning polypharmacological mechanisms of cardiovascular targets are very important to rationally design polypharmacological drugs.

### Explore the interactions between cardiovascular targets and genes

**Figure 3:**
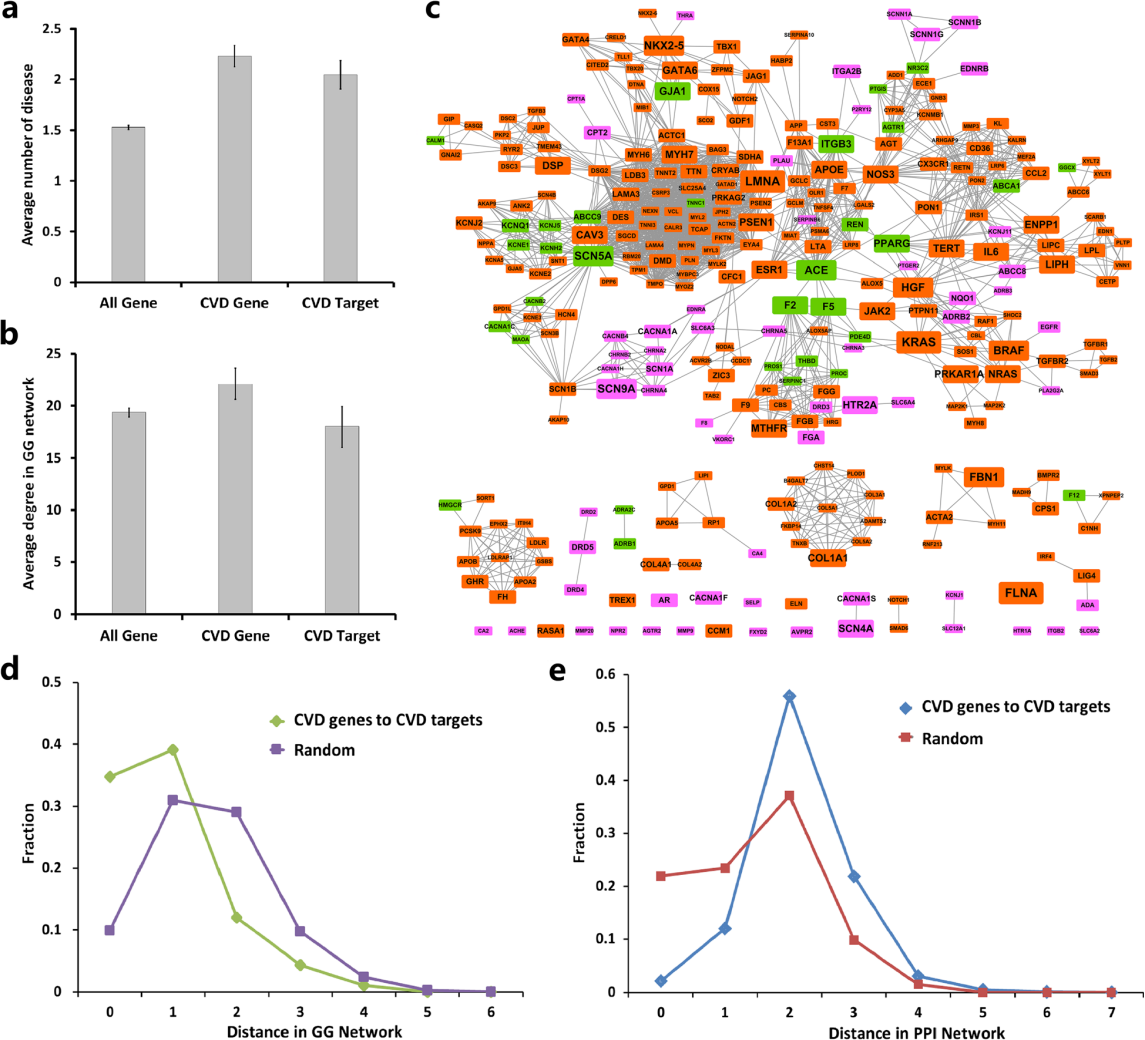
**Relationships between cardiovascular targets and genes. (a)** The average number of diseases associated with different gene classes from the gene-gene network (GG network): all genes, cardiovascular genes and genes that encode cardiovascular targets. **(b)** Average degree of different gene classes in the GG network. **(c)** The network only includes cardiovascular genes and genes that encode cardiovascular targets extracted from the overall GG network, in which two genes are connected if they are involved in the same disorder. This subnetwork shows that most genes that encode cardiovascular targets and cardiovascular genes gather into a complete network. Red, pink and green represent cardiovascular genes, genes that encode cardiovascular targets and overlapped genes, respectively. **(d)** Distribution of the shortest distances (green) between drug targets and disease genes in the GG network compared with that (tan) between random groups of genes. There is an enhancement at the distances 0 and 1. **(e)** Distribution of the shortest distances (yellow) between drug targets and disease genes in the GG network compared with that (blue) between random groups of genes.

To investigate the genetic feature of cardiovascular targets and detect the potential of cardiovascular genes to be therapeutic targets, it is necessary to analyze the relationships between cardiovascular targets and genes form a network perspective. We firstly compared the pleiotropy between them, which can be quantified by the number of disorders (node degree) corresponding to their mutants in the gene-disease network [32] (Additional file 1). The curated dataset contain 1,775 phenotypes and 3,039 associated genes (Additional file 1), of which 98 are cardiovascular phenotypes associated with 268 genes (Additional file 1). In addition, 111 disease genes encode the cardiovascular target proteins, of which 35 overlap the cardiovascular genes associated with 26 cardiovascular phenotypes (Additional file 1). As shown in Figure 3a, cardiovascular genes are on average involved in 2.2 ± 0.1 disorders, which is not significant different from that of cardiovascular targets (2.05 ± 0.13 disorders; *p* = 0.45, Wilcoxon rank-sum test). Both of them are significantly more than the average of all genes (1.5 ± 0.02 phenotypes; *p* < 10^−4^, Wilcoxon rank-sum test; Figure 3a). This indicates that cardiovascular targets and genes have similar degree of pleiotropy.

Data also show that these two groups stay close to each other in the disease gene-gene network (DGG network), where two genes connect with common diseases based on the global gene-disease associations (Figure 3c and Additional file 1). In the DGG network, 2,519 of 3,039 disease genes are connected to other disease genes, and 2,013 genes belong to a “giant component”. Ninety two genes that encode the cardiovascular targets and 249 cardiovascular genes are included in this network (Figure 3c). By measuring the minimum shortest distances between the targets and genes [10], the two groups are shown more intimate compared with those of the randomized expectations (Figure 3d, *p* < 10^−5^, Two-sample Kolmogorov-Smirnov test). Nearly 75% cardiovascular targets overlap with the cardiovascular genes or are in the first neighbors of them. Consistently, we also find that cardiovascular targets and genes have similar degree distributions (*p* > 10^−2^, Wilcoxon rank-sum test; Figure 3b) in the DGG network. As links in the DGG network represent the related phenotypic associations between two genes, the intimate relationships suggest that most of these targets are etiologically related factors, which provide further clues for disease understanding and treatment.

Previous work has shown that distinct genes that are related to same disorders tend to interact in a particular functional module [33]. Therefore, we believe that the genetic closeness between the cardiovascular targets and genes might have tendencies to gather together in the real cellular network. To test this, we quantify the relationships between the cardiovascular targets and genes in the human protein-protein interaction (PPI) networks by similar approach used in the DGG network (See Additional file 1). It is found there are 132 target proteins and 191 cardiovascular gene products in the PPI network. As expected, we observe a clear enrichment for cardiovascular targets to genes in the region of lower shortest distances compared with the randomized target groups of similar size (*p* < 10^−6^, Two-sample Kolmogorov-Smirnov test; Figure 3e). This is supported by the similar topological features between the targets and gene products in the PPI network (Additional file 1). This suggests that although many cardiovascular targets are not encoded by cardiovascular disease genes, they might also participate in the same physiological and pathological processes.

Finally, to validate this intimate relationship between cardiovascular targets and genes derived from the network properties, we also calculated their functional similarity distributions based on the GO-based semantic similarity (See Methods), and find significant similarity of GO terms between the two groups with respect to random controls, confirming their close relationships (Additional file 1; *p* = 0).

These results indicate that most cardiovascular drugs are etiology-specific agents that target the actual cause of the disease or etiologically related factors, which is a little unexpected as many types of CVDs are strongly influenced by non-genetic factors. More importantly, the intimate relationships between cardiovascular targets and genes could help understand the mechanism of action of cardiovascular targets and provide a direct evidence for target identification from the cardiovascular genes [28].

### Explore the relationships between cardiovascular disorders

During these years, huge efforts have been devoted to the use of networks (disease network) to integrate different genetic, proteomic, metabolic and phenotypic datasets to elucidate the entangled origins of many diseases [34-36]. Here, to examine the relationship between cardiovascular disorders, we generated a gene disease-disease network (GDD network; Additional file 1), which is transformed by connecting two disorders if they are associated with the same gene based on the gene-disorder associations, The GDD network consists of 1216 disorder nodes connected by 2858 links, where the largest component comprises 942 nodes and 2596 links. Of 98 cardiovascular disorders in the OMIM, 64 have at least one link to other disorders and are included in this network. The number of genes involved in cardiovascular phenotypes decreases rapidly (Additional file 1): most diseases are related to few genes, whereas some related to dozens of genes, such as cardiomyopathy (45 genes), coronary artery disease (15 genes) and myocardial infarction (15 genes). The gene distribution may correlate with the complexity of each disorder in some extent. Generally, Mendelian disorders such as Marfan syndrome are mostly derived from mutations in one or several genes, whereas complex disorders such as myocardial infarction are related to multiple genetic determinants. In addition, the number of degree of the cardiovascular disorders in the GDD network display a broad distribution (Additional file 1) and most of them are connected to more than one disease, especially a few disorders such as cardiomyopathy (degree = 43), Noonan syndrome (degree = 24) and myocardial infarction (degree = 24) are connected to a large number of distinct disorders. On average, the degree of cardiovascular disorders (6.2 ± 0.7 disorders) is significantly bigger than that of the network average (4.7 ± 0.2 disorders; *p* = 0.0009, Wilcoxon rank-sum test; Additional file 1). This prominence of the highly connected disorders should mainly arise from the mutations that are involved in multiple disorders.

**Figure 4:**
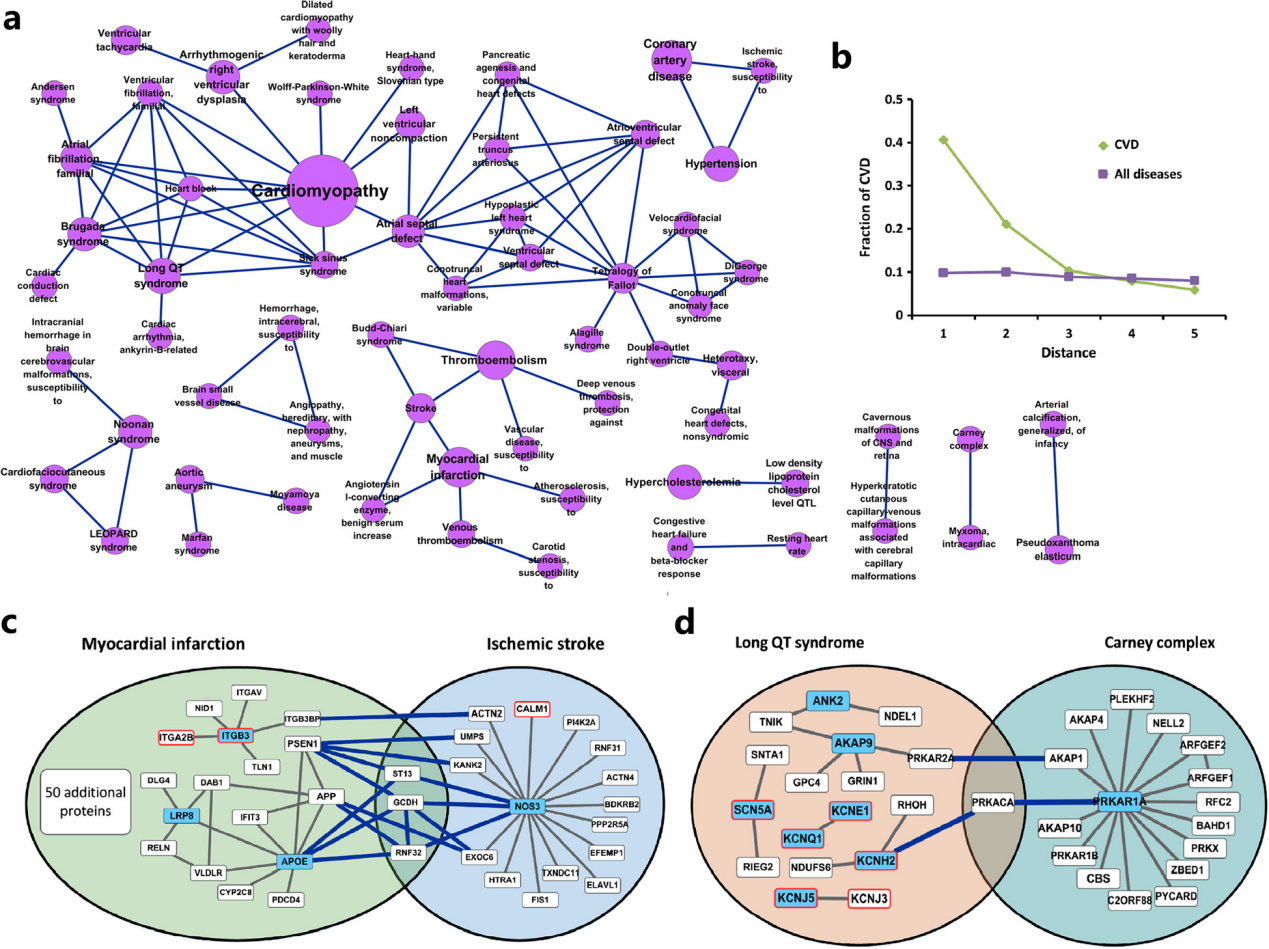
**Relationships between different cardiovascular disorders. (a)** Selected networks from the gene disease-disease network (GDD network). This network is composed by cardiovascular disorders separated from the GDD network, where each node corresponds to a disorder and two disorders are linked if there is a gene involved in both. The size of each node corresponds to the number of genes that are implicated in this disease. This network shows many cardiovascular disorders tend to related to other cardiovascular disorders. **(b)** Fraction of cardiovascular disorders starting from either a cardiovascular disorder or a random disorder in the GDD network with respect to distance. This figure quantitatively validates the bias of cardiovascular disorders toward clustering in the GDD network. Two examples of cardiovascular disease pairs with significant protein-protein interactions: **(c)** Ischemic stroke and myocardial infarction (PPIs = 15; p << 0.01, z-test), and **(d)** Long QT syndrome and Carney complex (PPIs = 3; p = 1.88E-57, z-test). The blue-filled rectangles are cardiovascular disease genes. The rectangles with red border are cardiovascular targets. The blue-filled rectangles with red border are both cardiovascular targets and genes. Other proteins are the neighbors of cardiovascular genes. See Additional file 1for the global cardiovascular disease modular network.

Most cardiovascular disorders are visibly clustered in the network (Additional file 1; see Figure 4a for cardiovascular disorder associations). To quantify this, we measured the fraction of cardiovascular disorders with the reference to the distance from an origin disease node in the network. If these disorders are not clustered in certain regions, starting from one cardiovascular disorder would not be different from a random node. Instead, we observed a surprising enrichment in the first and the second neighbors for an origin node of CVD (Figure 4b), indicating a strong trend of concentrating cardiovascular disorders in the GDD network. This means that most cardiovascular disorders share genetic origins with each other.

We further examined the extent to which cardiovascular disorders aggregate in the GDD network. Firstly,we attempted to extract all types of these disorders by disconnecting them from the whole network, and found that they form many isolated sub-networks (Figure 4a). The largest sub-network contains 31 disorders, most of which are cardiogenic traits, such as cardiomyopathy, long QT syndrome and atrial fibrillation. The second largest cluster includes the major vascular diseases, such as stroke, myocardial infarction and atherosclerosis. Following on are Mendelian disorders that form small isolated networks consisting of 1 ~ 3 nodes. These results indicate that the isolated networks may represent relatively independent pathological mechanisms of cardiovascular disorders. This reminds us that drugs used for one disease might also affect other disorders of the sub-network. For example, an anticoagulant, acenocoumarol, can be used to treat various vascular diseases including deep vein thrombosis, ischemic attack, myocardial infarction and thromboembolism. The above analysis further suggests that some other anticoagulants, such as defibrotide, prasugrel and sulodexide, might also have potentials to treat blood coagulation-caused CVDs.

We then reconnected these cardiovascular disorders to their direct neighbors (non-cardiovascular disorders) in the GDD network. Surprisingly, nearly 90% (61 disorders) of these disorders are re-accumulated into a complete network (Additional file 1), confirming the close associations between different CVD-associated sub-networks. In addition, there are 197 non-cardiovascular disorders (70.4% of all disorder node) in this reconnected network, most of which are enriched by some common cardiovascular disorders such as cardiomyopathy and myocardial infarction. This prominence of non-cardiovascular disorders can be partly attributed to the involvement of the cardiovascular factor in various disease conditions [37], such as diabetic nephropathy, pheochromocytoma and diabetes mellitus. This also prompts us to seek drugs for CVD treatment from those drugs that are applied for treating other diseases. For example, the antidepressant agent, paroxetine, has been under evaluation in clinical trials for its potential value in preventing heart attacks (www.clinicaltrials.gov).

### Identify the molecular connections between cardiovascular disorders

GDD network that covers the discrete genetic information might not be sufficient to explain the molecular processes for disease associations. Here, we further try to explore the molecular connections between different cardiovascular disorders based on their cellular modules encoded by PPI network. Firstly, we created the disease modules for each disorder using the interaction patterns of their associated gene products in the PPI network [20] (See Methods). This results in a highly interconnected disease modular network, which includes 72 disease modules (Additional file 1). The figure shows that genes in each disorder have a strong tendency to interact with each other at the protein level. For each pair of diseases, we assessed their molecular connections by measuring the significance of their shared PPIs by randomization tests of the resulting network [38] (the procedure is described in detail in Methods). The full sets of assessments include 2,556 disease pairs, of which 227 (8.88%) share significantly more protein-protein interactions compared with that of a random control (Additional file 1; *p* < 0.01, z-test). This information can assist us in identifying new molecular connections between disorders alongside their common genetic origins. For example, except for the common disease gene JAG1 between Tetralogy of Fallot and Alagille syndrome, the two diseases interact through 5 PPIs with a *p* value < < 0.01 in the network, including JAG1 – NOTCH1, JAG1 – NOTCH2, JAG1 – NOTCH3, DLL1 - NOTCH2 and DLL1 – NOTCH3. All these five PPIs are all involved in the medication of Notch signaling pathway that functions in cell-fate decisions during hematopoiesis and early and late stages of mammalian cardiovascular development. Indeed, abnormalities of this pathway has been proved to be implicated in both Tetralogy of Fallot and Alagille syndrome and the comorbidity of the two diseases has been well known to medical community [39,40]. More interestingly, we find several disease pairs can be linked by only the cellular-level interactions. For example, ischemic stroke and myocardial infarction share 15 PPIs (Figure 4c; *p* < < 0.01, z-test) in the network, although there are no common disease genes between them. Specifically, some shared PPIs such as APP - EXOC6 functions in the amyloid formation, which has been known to be involved in both stroke and myocardial infarction [41]. These connections are also supported by the well-known relevance of the two diseases in clinic [42,43]. Similarly, we also observed significant interactions between long QT syndrome and Carney complex (*p* < 10^−57^, z-test). As shown in Figure 4d, the common three PPIs and their associated genes between the two disease modules are all involved in the protein kinase A (PKA) signaling pathway. As we know, this pathway is the major route for channeling the second messenger cAMP signal [44] and has been proved to be implicated in both long QT syndrome [45] and Carney complex [46]. Finally, many unknown diseases pairs are observed based on the molecular connections, such as Noonan syndrome and Carney complex (shared PPIs = 5, *p* < 10^−17^), LEOPARD syndrome and Brugada syndrome (shared PPIs = 4, *p* < 10^−19^), Cardiofaciocutaneous syndrome and Aortic aneurysm (shared PPIs = 4, *p* < 10^−149^). These results could potentially provide insights into the disease pathogenesis and the design of novel therapies for CVD. A more detailed description of these disease pairs are provided in the Additional file 1.

## Discussion

CVD, as a complex disease, is the consequence of a collection of deleterious effects from interactions involved multiple genetic and environmental origins. In recent years, systems-based approaches have nearly become a consensus for explore cardiovascular problems from disease pathogenesis to therapy [11,47,48]. However, the corresponding studies, especially from quantitative perspective, are still insufficient. Here, we use the concepts of systems pharmacology to integrate publicly available CVD-associated data and provide a complete framework to quantify the underlying relationships between cardiovascular drugs, targets, genes, and diseases. An online database CVDSP (http://sm.nwsuaf.edu.cn/lsp/cvdsp.php) is developed to allow researchers to access the underlying information of CVD systems biology and pharmacology in a user-friendly manner (See Additional file 1). CVDSP is a comprehensive annotated resource that combines all available information of cardiovascular drugs with therapeutic protein targets, cardiovascular disorder-to-gene associations, as well as the corresponding networks. Studies based on this database would help deepen our understanding of the mechanisms of cardiovascular drug actions and disease complexity and to facilitate target discovery and drug design.

In the context, we mainly focus on three areas that are critical to cardiovascular pharmacology derived from the emerging properties of these networks.

(i) The cardiovascular drug-target interaction. We examine the drug-target network and the derivative drug-drug and target-target network and generate a rich network of polypharmacological interactions between the cardiovascular drugs and their targets. These results indicate the promiscuous nature of cardiovascular drugs and prompt the exploration of drugs that target multiple proteins and combination therapies for CVD [11], however, the impact of the nonselectivity-caused side effects should not be under estimated. The promiscuous drug information (listed in CVDSP) will provide important clues concerning targets for drug discovery. Those known multitarget drugs can be used as lead or reference compounds to design new drugs with a specific multi-target profile to achieve a desired polypharmacology [49].
  The drug-target interactions are also visualized beyond the incorporation of the approved drugs and primary therapeutic targets. For example, an extended drug-target network including the experimental medicines (drugs in the pipeline or not yet approved by the FDA) for CVD therapy and their therapeutic targets are used to quantify trends in exploitation of novel drugs and targets (see details in Additional file 1). In addition, the extended drug-target network that expands those drugs and targets irrelevant with CVD will further prioritize connections between the non-cardiovascular drugs and either therapeutic or unwanted cardiovascular effects, resulting in identification of novel potential drug-target interactions [50,51] (see details in Additional file 1: “The third layer of drug-target network”).
(ii) The relationships between cardiovascular targets and genes. The cardiovascular protein targets and genes tend to intimately interact with each other in the gene and interactome networks with similar topological properties. This close relationship is also confirmed by their functional similarity distributions (*p* = 0; Additional file 1). This will facilitate our understanding of the molecular mechanisms of CVD treatment and firm our beliefs to identify the druggable target genes. For example, we can rank all these genes using the enrichment of known cardiovascular targets in their first-order interaction network, to identify potential target candidates [38], such as F2, F5 and PROC, which have been demonstrated involved in thromboembolism disease.
(iii) The associations between cardiovascular disorders. GDD network share more etiological commonalities with each other rooted in the global disease-disease networks. Cardiovascular disease module analyses indicate that most cardiovascular disorders have significant molecular connections among them (Additional file 1). Previous studies have shown that distinct disease phenotypes with complex interdependencies among cellular components usually have many functional and causal relationships [28,52,53]. Therefore, the systematic identification of such network-based dependencies among cardiovascular disorders offers a sufficient resolution and specificity for etiologic heterogeneity and clinical treatment of CVD. Indeed, huge efforts have been devoted to the use of disease networks (diseasome) to integrate different genetic, proteomic, metabolic and phenotypic datasets to elucidate the entangled links of diseases [13,34,54]. Uncovering such links between diseases could help understand how and why different disorders are linked at the molecular level. The relevance of conditions that is culled from the diseasome offer insights into disease classification, prevention, diagnosis, and treatment. Diseasome-based approaches could also aid drug discovery, in particular when it comes to the use of approved drugs to treat molecularly linked diseases. For the common genes or proteins shared by diseases shown in the disease-module network, drugs designed for one of the disorders may also be used for the other. For example, ramipril that initially developed for hypertension also treat myocardial infarction and stroke. Similarly, phenindione can be used for atrial fibrillation and cardiomyopathy, and triflusal for thromboembolism and stroke (Additional file 1). In addition, we generated a drug disease-disease network (Additional file 1) by connecting any two diseases which can be treated with the same drug. Similar to the drug-target analysis (See details in Additional file 1), we can also suggest novel drug uses (drug repositioning) according to these close disease pairs in the drug disease-disease network which was built by connecting any two diseases treated with the same drug. Given the shared medications between disease pairs in this network, especially a high number of drugs against both disease classes, drugs used for only one of the two may also be used for the other (See details in Additional file 1: Cardiovascular drug-indication associations).

## Conclusions

In summary, our paradigm mainly involves the key factors including drug, target, gene and disease underlying cardiovascular systems biology and pharmacology. Indeed, many other factors such as environmental stress, epigenetic modifications and invasion of pathogens also contribute to diseases. Incorporating these factors will further improve the coverage and significance of the networks [55]. However, presently, it is still difficult to combine all these together for deep analysis due to the lack of sufficient and high-quality data. In addition to the static network analysis, we hope the dynamic networks such as metabolic and transcriptional network, which are also important, should be integrated in the follow-up studies [56]. As methodologies evolve, the systems pharmacology is believed to provide a complete picture that allows us to appreciate the networked nature of human diseases, to design new pharmacological models and then to guide the experiments to new drug discovery and disease treatment.

## Additional file

Additional file 1:
**Insights from systems pharmacology into cardiovascular drug discovery and therapy.** Additional files are available online. Especially, Supplementary Datasets are available at http://sm.nwsuaf.edu.cn/lsp/load_intro.php?site=cvdsp&id=48.

## Abbreviations

CVD: Cardiovascular disease
GPCRs: G protein-coupled receptors
ATC: Anatomical Therapeutic Chemical
DGG network: Disease gene-gene network
PPI: Protein-protein interaction
GDD network: Gene disease-disease network
