# Supplementary material for: Insights from systems pharmacology into cardiovascular drug discovery and therapy
Source: BMC Syst Biol. 2014 Dec 24;8:141. doi: 10.1186/s12918-014-0141-z (PMC4297424; doi:10.1186/s12918-014-0141-z)
Supplement: Additional file 1: — Insights from systems pharmacology into cardiovascular drug discovery and therapy. Additional files are available online. Especially, Supplementary Datasets are available at http://sm.nwsuaf.edu.cn/lsp/load_intro.php?site=cvdsp&id=48. [file 12918_2014_141_MOESM1_ESM.doc]

**Supplementary information:**

**Insights from systems pharmacology into cardiovascular drug discovery and therapy**

**The first layer of drug-target network**

The first layer of drug–target network (FL_DT network) was built based on this data including 254 drugs and 206 protein targets by connecting the approved cardiovascular drugs with their corresponding cardiovascular targets (Figure 2a). The overall network shows 701 drug-target connections. The distribution of drug node degrees shows that most drugs are subject to a few targets, while several have multiple targets even up to 20 (Figure 2b). The average number of target proteins per drug is 2.8. Likewise, many proteins are also targeted by multiple drugs (Figure 2c). In the FL_DT network, 198 drugs (~78% of the total) and 165 targets (~80% of the total) compose the largest connected component of the network, i.e., the so-called giant component (Figure 2a), indicating the interconnectedness between the cardiovascular drugs and their targets. Starting from this graph, we generate two biologically relevant network projections. In the “target–target (TT) network” (Supplementary Figure S2), nodes represent targets, and two protein targets are connected to each other if they share at least one drug. In the 206 targets, 200 have at least one link to other targets, that is, they share drugs with other targets. In the “drug–drug (DD) network”, nodes represent drugs, and two drugs are connected if they are associated with the same protein target (Supplementary Figure S1). 243 out of 254 drugs are connected to other drugs, of which, most drugs (198) are integrated into a large complete network with 1424 interconnections. We colored the network nodes with the annotation concerning drug ATC code and target functional class. It is evident that specific drug classes tend to have their members clustered together with common targets between each other, and their targets are mostly within the same gene family, such as the concentrated community of beta blockers and their targeted adrenergic receptors.

**The second layer of drug-target network**

The second layer of drug-target network (SL_DT network) was constructed by attaching the experimental medicines (drugs in the pipeline or not yet approved by the FDA) for CVD therapy and their therapeutic targets on the existing components of the FL_DT network (Supplementary Dataset S2; supplementary Figure S3). In total, there are 108 experimental cardiovascular drugs and 64 corresponding targets to be collected from TTD (Supplementary Dataset S2). Of them, twenty two chemical entities (~39% of all the experimental cardiovascular drugs) are “follow-on” drugs that interact with 22 established targets. Most targets (~66% of all introduced targets) of the experimental medicines have been categorized to previously un-targeted proteins and form several small isolated networks. It suggests that many cardiovascular drugs tend to be developed based on the previous unexploited targets.

**The third layer of drug-target network**

At last, the third layer of drug-target network (TL_DT network) was created to assess the capacity to discover new knowledge of potential cardiovascular drugs and targets at systems level. The TL_DT network expands those drugs and targets without the well-established associations with CVD but relevant to cardiovascular targets and drugs from the FL_DT network, respectively. Inclusion of these drugs and targets increases the size of the “giant component” of FL_DT network to 1028 (Supplementary Dataset S3; supplementary Figure S4). Interestingly, these drugs and targets have integrated some previous isolated networks into the “giant component”, such as ACE inhibitors and HMGCR inhibitors (supplementary Figure S4). A further inspection of this network might be beneficial to new drug repositioning and disclose novel molecular mechanisms for CVD therapy.

Corresponding to cardiovascular targets, there are 579 drugs that are not labeled as cardiovascular drugs. The non-cardiovascular drugs show similar target distributions to the known cardiovascular drugs, and the average number of drug targets per non-cardiovascular drug is also 2.8. We also classified these non-cardiovascular drugs according to the ATC code and found that they include all other anatomical main groups except cardiovascular drug types (Supplementary Dataset S3), indicating that CVD has a high concentration of the shared targets with other disease classes. This should be partly due to the involvement of the circulatory system in various disease conditions^1^. Out of the non-cardiovascular drugs, drugs for nervous system are the most common class, comprising ~33% of all non-cardiovascular drugs. They are followed by agents for a), alimentary tract and metabolism, b), respiratory system, c), musculo-skeletal system, d), genitourinary system, e), skin, f), sensory organs, g), malignant and immune disease, h), endocrine system, i), blood and blood forming organs and j), infections and infestations, which comprise ~13%, ~11%, ~7%, ~6%, ~5%, ~5%, ~4%, ~1%, ~1%, and ~1% of all the non-cardiovascular drugs, respectively. These non-cardiovascular drugs might produce therapeutic or adverse cardiovascular effects mediated by their own CVD-associated targets. For example, many atypical antipsychotic drugs such as quetiapine might increase incidence of cerebrovascular adverse events (eg. stroke, transient ischemic attack)^2^. In contrast, some other drugs such as an antidepressant (paroxetine) have been under evaluation in clinical trials for its potential value in preventing heart attacks (www.clinicaltrials.gov). Actually, this attempt to find novel indications of the approved drugs which differs from their original intent will be beneficial to new drug repositioning^3^.

Corresponding to the cardiovascular drugs, there are 51 targets which are still not related to CVD (Supplementary Dataset S3; Supplementary Figure S4). However, we cannot exclude the potential associations of these non-cardiovascular targets with CVD. A further inspection of them might disclose new molecular mechanisms for CVD therapy. In addition, these non-cardiovascular proteins are targeted by 57 cardiovascular drugs that hold only ~28% of all cardiovascular drugs, indicating that most of the cardiovascular drugs are specifically designed for CVD.

**Cardiovascular drug-indication associations**

The practice of a cardiovascular medicine is largely governed by a clinical phenotype based approach. However, since the traditional way to classify diseases is according to the observational results between pathological analysis and clinical syndromes via a reductionist approach^4^, it is not straightforward to figure out whether the contemporary drug treatments can manifest the phenotypic interdependencies or disease cooccurrences. To answer this question, we manually mapped the approved cardiovascular drugs to diseases by searching for disease keywords in the ‘indications’ field of the drug information obtained from the DrugBank database, first automatically and then by validating resulting associations manually. Those diseases were filtered for vague (e.g. rapid detoxification), degree (e.g. mild hypertension and moderate hypertension) and synonym (e.g. cardiac dysrhythmias and arrhythmia) terms, whereas the concept terms that are the child terms of one higher hierarchical term (e.g. atrial fibrillation and arrhythmia) and general term (e.g. cardiovascular disease) are not removed. Finally, 614 drug-disease associations (Supplementary Dataset S8) were created, that contain 166 diseases (60 cardiovascular disorders and 106 non-cardiovascular disorders). From the drug-disease associations, we further generated a drug disease-disease network (DDD network, Supplementary Figure S12) by connecting any two diseases which can be treated with the same drug.

If most diseases are treated with distinct drugs, then the DDD network would consist of many isolated nodes corresponding to specific disorders or small clusters of a few closely related disorders. On the contrary, the resulting DDD network possesses high connections between different diseases (Supplementary Figure S12). Out of 166 disorders, 158 connect to each other by 694 links, and 144 disorders form a “giant component” with 681 edges. The distribution of the drug number associated with CVD shows that a few CVD types, such as hypertension (101 drugs), heart failure (40 drugs) and angina (27 drugs) possess more medications than other types (Supplementary Figure S13a). Moreover, the distribution of the drug number for these cardiovascular disorders is well correlated to the distribution of the disorder degrees in the DDD network. Most CVD types with high concentration of therapies also tend to be network hubs connecting to a large number of other disorders (both cardiovascular and non-cardiovascular) (Supplementary Figure S13b). This correlation is reasonable as these hub disorders are usually epidemiologically prevalent and tend to be concomitant with many other diseases^5^. For example, hypertension, as the hub disorders in the DDD network, is the most prevalent risk factor for many disorders such as coronary heart disease, angina, and heart failure. And the co-emergence of these diseases is well captured in the DDD network (Supplementary Figure S12).

The number of the non-cardiovascular disorders (102 disorders) is much bigger than that of the cardiovascular disorders (56 disorders) in the DDD network. This suggests that most cardiovascular disorders do share highly similar medications not only between themselves but also from many other disease classes. This can be partly attributed to the involvement of the cardiovascular factor in various disease conditions^6^, especially diseases linked to more CVD nodes, such as diabetic nephropathy (linked to 8 cardiovascular disorders), pheochromocytoma (linked to 5 cardiovascular disorders) and diabetes mellitus (linked to 4 cardiovascular disorders). Moreover, similar to the drug-target analysis in section 1, we can also suggest novel drug uses (drug repositioning) according to these close disease pairs in the DDD network. Given the shared medications between disease pairs in this network, especially a high number of drugs against both disease classes, drugs used for only one of the two may also be used for the other. For example, there are 101 drugs used for hypertension, 40 drugs for heart failure, and 25 drugs for both diseases (Supplementary Dataset S9). We suggest that the other 76 drugs for hypertension may treat heart failure and the other 15 drugs for heart failure can be used for hypertension. To validate these suggestions, we have tested whether these suggested novel drug uses have been already evaluated in clinical trials. Surprisingly, we found 5 of the 15 drugs for heart failure have been suggested for hypertension and 14 of the 76 drugs for hypertension have been suggested for heart failure in clinical trials. Indeed, this treatment-based approach has been successfully used for drug repositioning^7^.

**References**

1. Zheng C, Han L, Yap C, Ji Z, Cao Z, Chen Y. Therapeutic targets: progress of their exploration and investigation of their characteristics. *Pharmacol Rev* 2006, 58(2): 259-279.

2. Schneider LS, Dagerman KS, Insel P. Risk of death with atypical antipsychotic drug treatment for dementia. *JAMA* 2005, 294(15): 1934-1943.

3. Ashburn TT, Thor KB. Drug repositioning: identifying and developing new uses for existing drugs. *Nat Rev Drug Discov* 2004, 3(8): 673-683.

4. Goh K-I, Cusick ME, Valle D, Childs B, Vidal M, Barabasi A-L. The human disease network. *Proc Natl Acad Sci U S A* 2007, 104(21): 8685-8690.

5. Loscalzo J, Kohane I, Barabasi A-L. Human disease classification in the postgenomic era: a complex systems approach to human pathobiology. *Mol Syst Biol* 2007, 3(1).

6. Paul M, Mehr AP, Kreutz R. Physiology of local renin-angiotensin systems. *Physiol Rev* 2006, 86(3): 747-803.

7. Chiang AP, Butte AJ. Systematic evaluation of drug–disease relationships to identify leads for novel drug uses. *Clin Pharmacol Ther* 2009, 86(5): 507-510.

**Supplemental Figures**

**
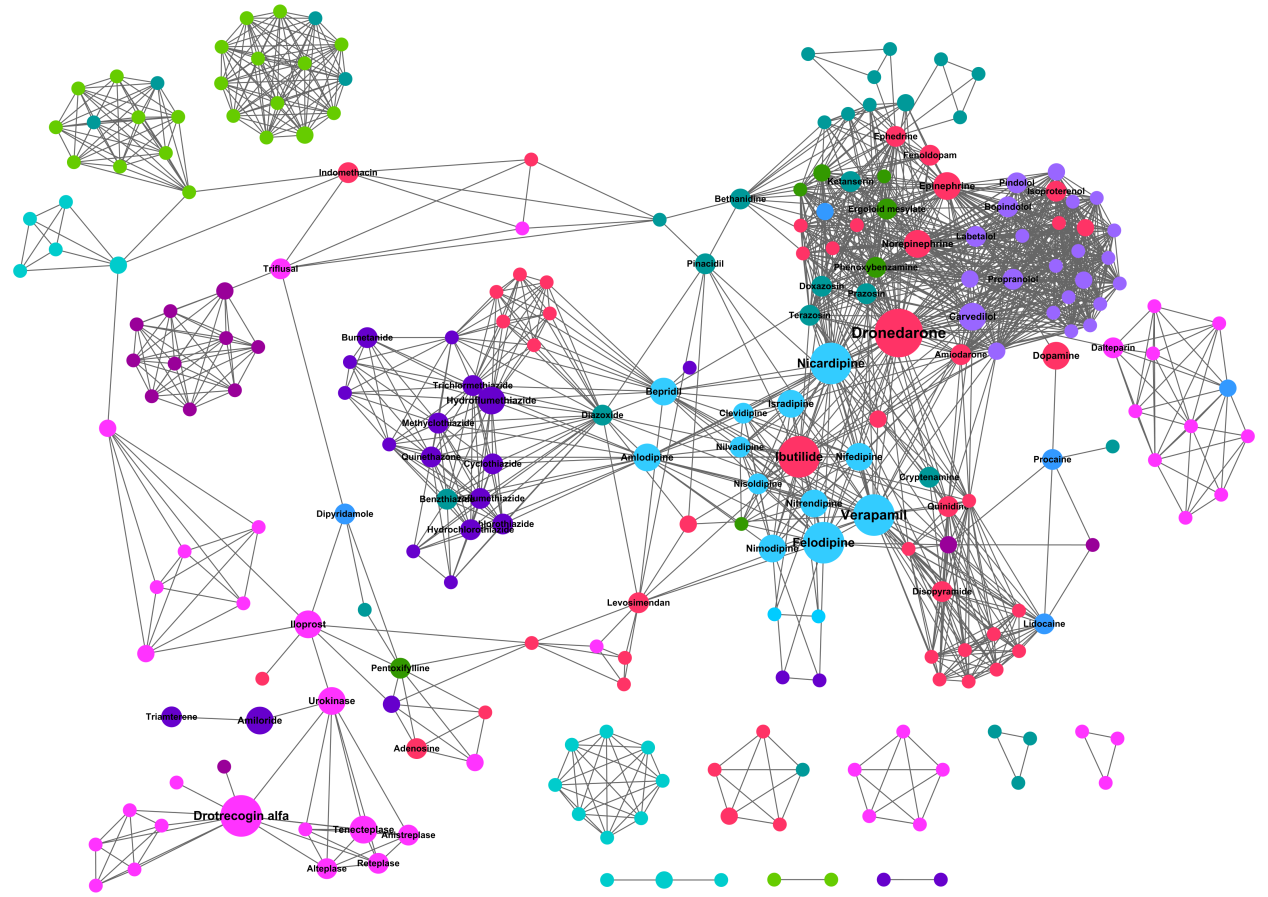
**

**Supplementary Figure S1. The drug–drug network.** In the drug-drug network, each node corresponds to a cardiovascular agent, and each node is colored according to their Anatomical Therapeutic Chemical (ATC) Classification. The size of each node is proportional to the number of proteins targeted by the drug.

**
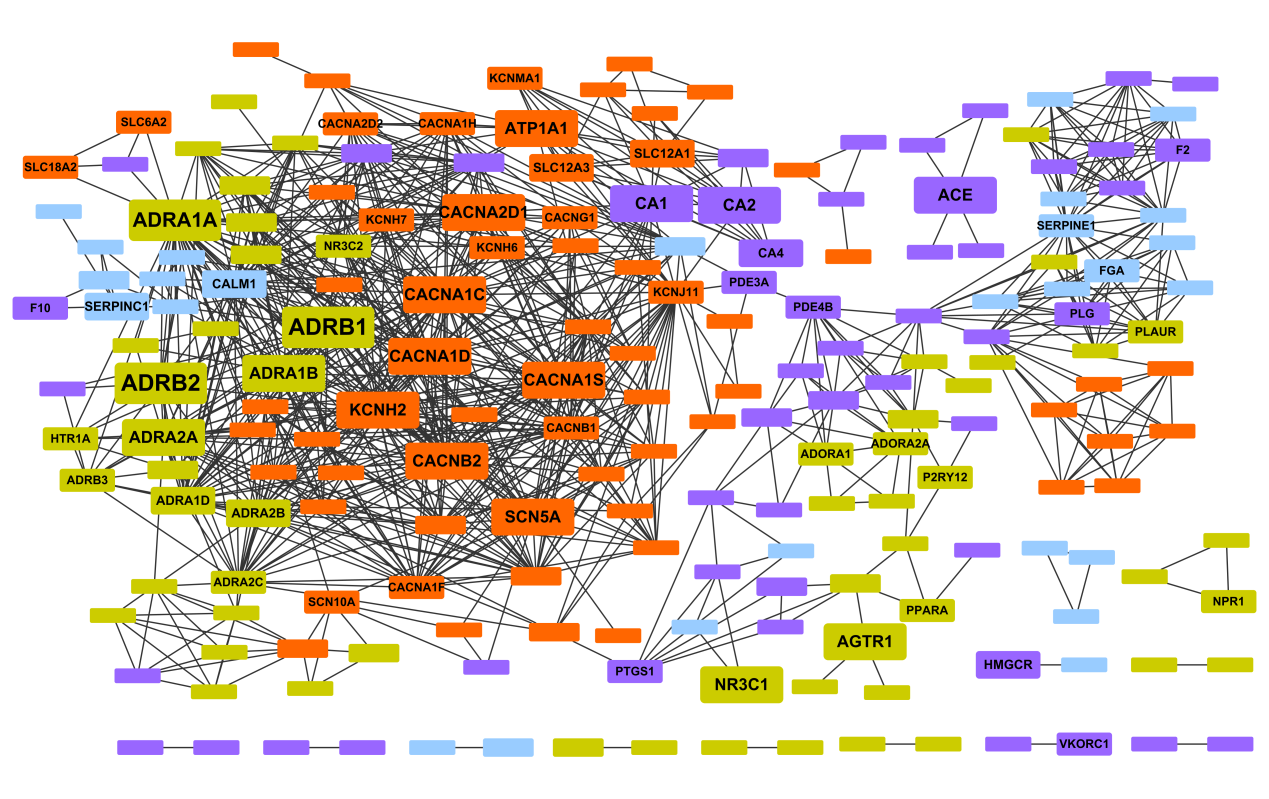
**

**Supplementary Figure S2. The target–target network.** In the target-target network, each node is a protein, two proteins being connected if they are targeted by the same drug. The size of each node is proportional to the number of drugs targeting the gene. The nodes are colored according to their functional families.

**
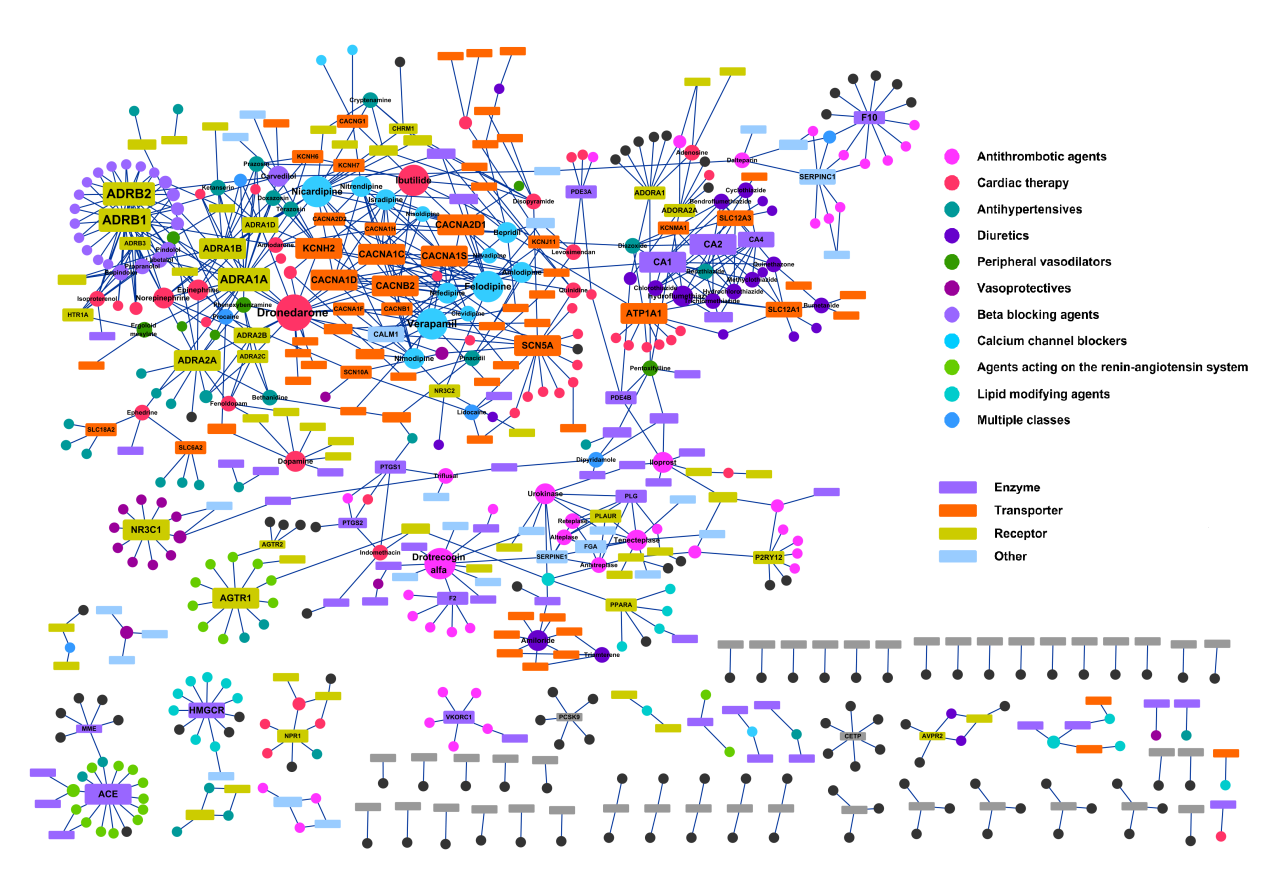
**

**Supplementary Figure S3. The second layer of drug–target network (SL_DT network).** The SL_DT network was generated by including the experimental medicines (drugs in the pipeline or not yet approved by the FDA) for CVD therapy and their therapeutic targets into the first layer of drug-target network (FL_DT network). In total, there are 108 experimental cardiovascular drugs and 64 corresponding targets to be collected from TTD. Nodes represent drugs (shown as circles) and targets (shown as rectangles). A link is placed between a drug and a target node if the protein is a known target of the drug. The size of the drug (protein) node is proportional to the number of the relevant targets (the number of the relevant drugs). Approved drugs are colored according to their Anatomical Therapeutic Chemical (ATC) Classification, and corresponding targets are colored according to protein family obtained from the Uniprot database. Experimental drugs and targets are grayed. The network was viewed in Cytoscape and analyzed using local Python scripts.


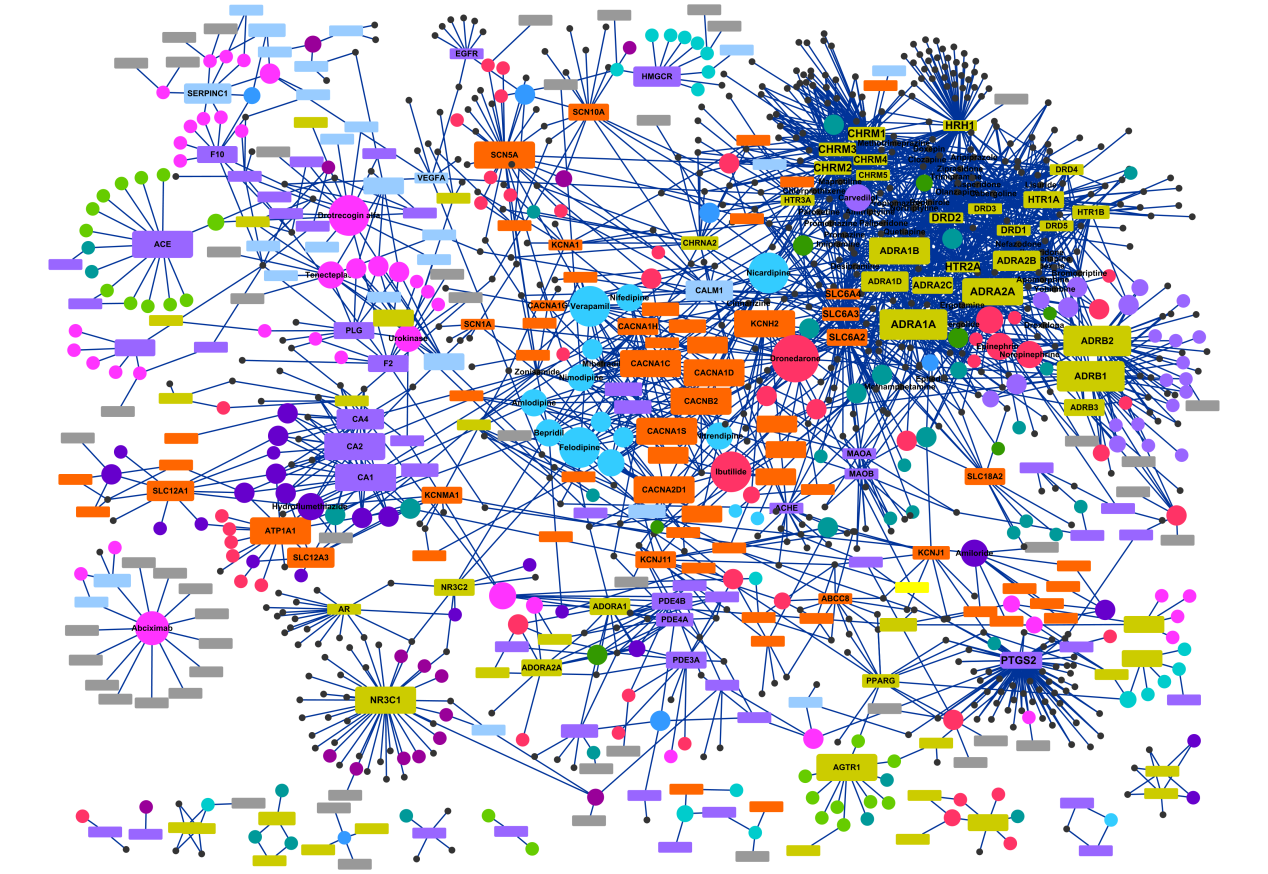


**Supplementary Figure S4. The third layer of drug–target network (TL_DT network)**. The TL_DT network was generated by expanding those drugs and targets without the well-established associations with CVD but relevant to cardiovascular targets and drugs from the first layer of drug-target network (FL_DT network), respectively. In total, there are 579 non-cardiovascular drugs and 51 non-cardiovascular targets. Nodes represent drugs (shown as circles) and targets (shown as rectangles). A link is placed between a drug and a target node if the protein is a known target of the drug. The size of the drug (protein) node is proportional to the number of the relevant targets (the number of the relevant drugs). Approved drugs are colored according to their Anatomical Therapeutic Chemical (ATC) Classification, and corresponding targets are colored according to protein family obtained from the Uniprot database. Non-cardiovascular drugs and targets are grayed. The network was viewed in Cytoscape and analyzed using local Python scripts.


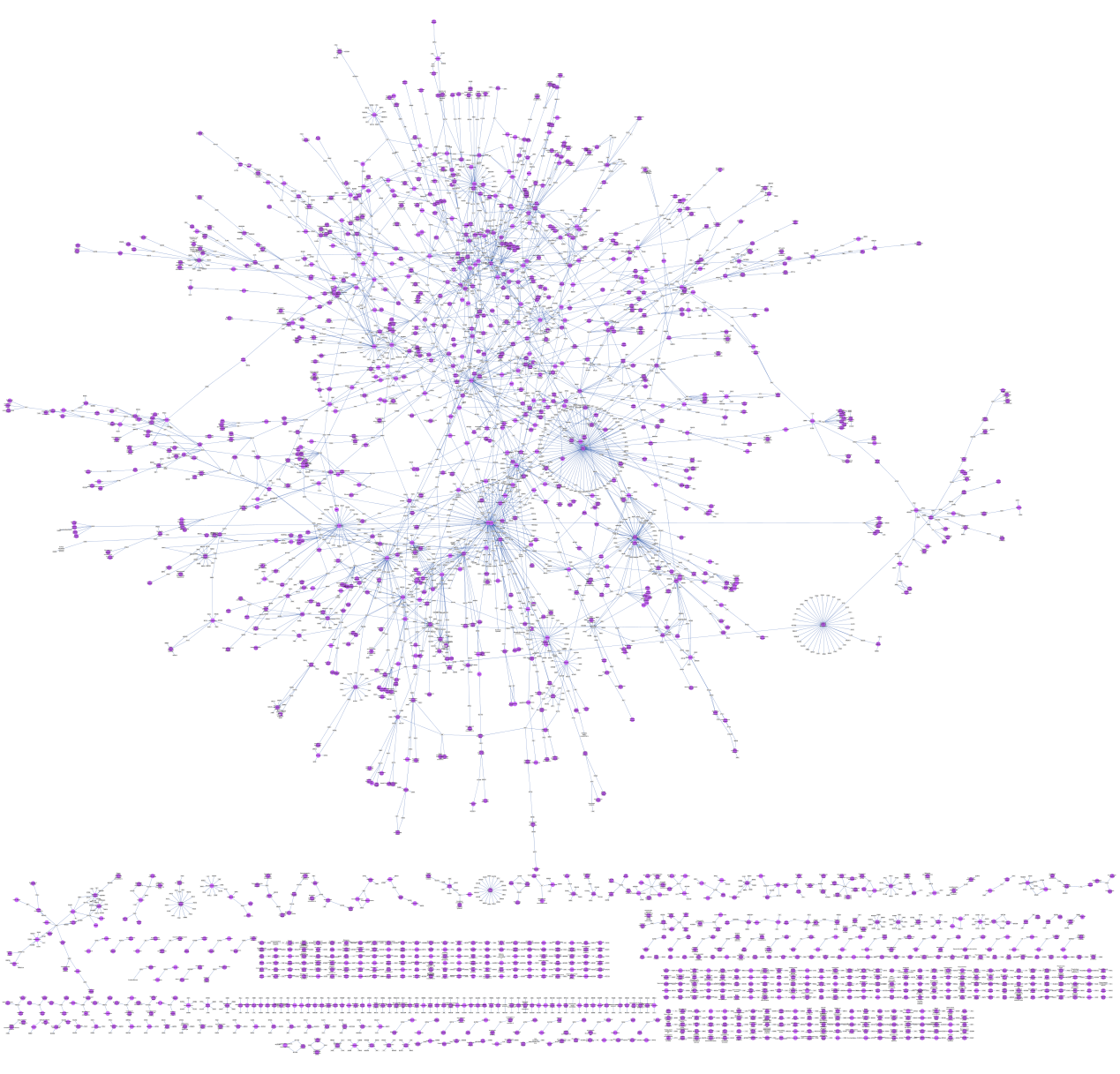


**Supplementary Figure S5. Gene-disease network.** In the human gene-disease network, circles and rectangles correspond to disorders and disease genes, respectively. A link is placed between a disorder and a disease gene if mutations in that gene lead to the specific disorder.

**
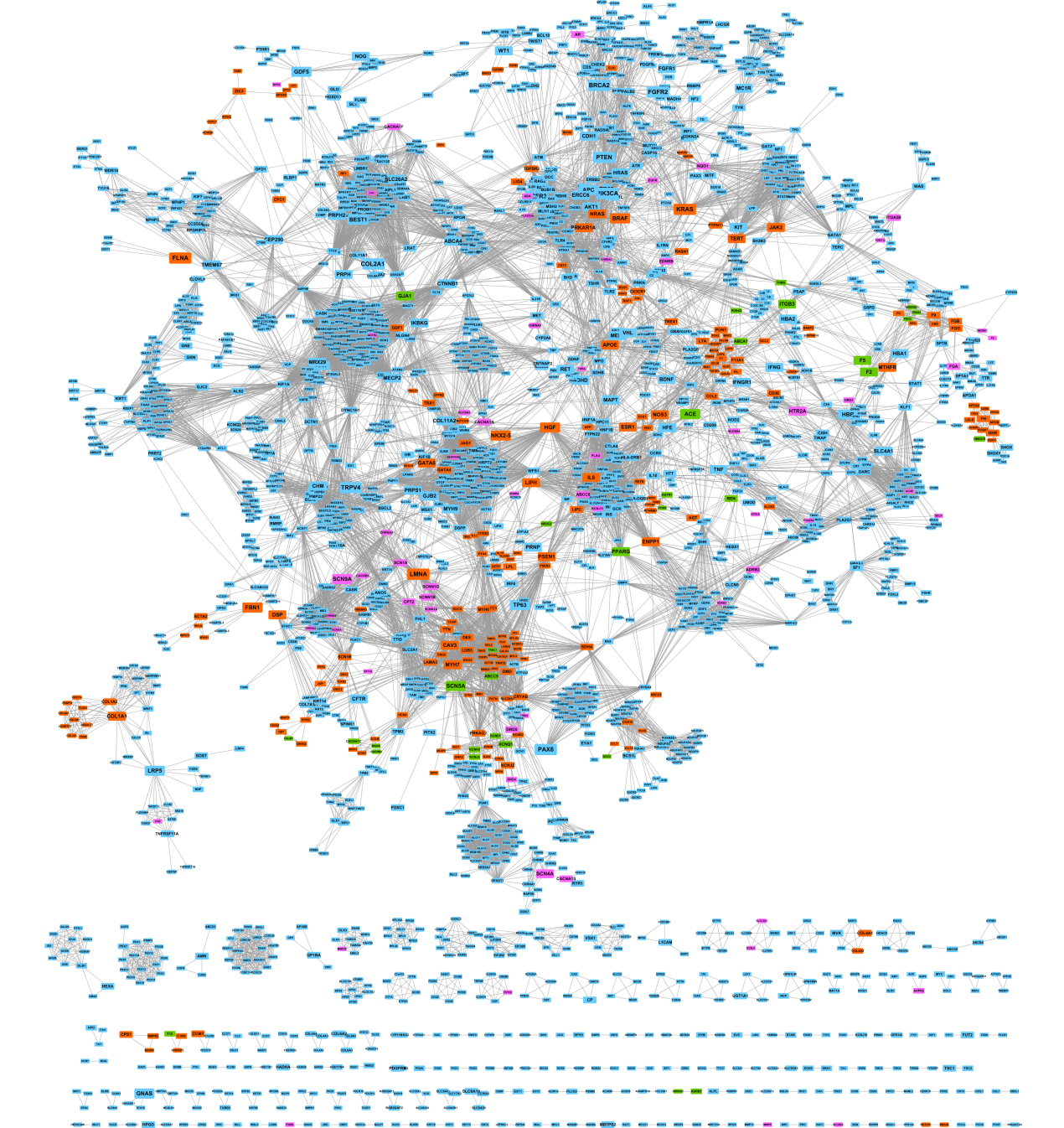
**

**Supplementary Figure S6. Disease gene-gene network (DGG network).** In the DGG network, each node is a gene and two gene nodes are connected if they are involved in the same disorder. Red, pink and green represent cardiovascular genes, genes that encode cardiovascular targets and overlapped genes, respectively.


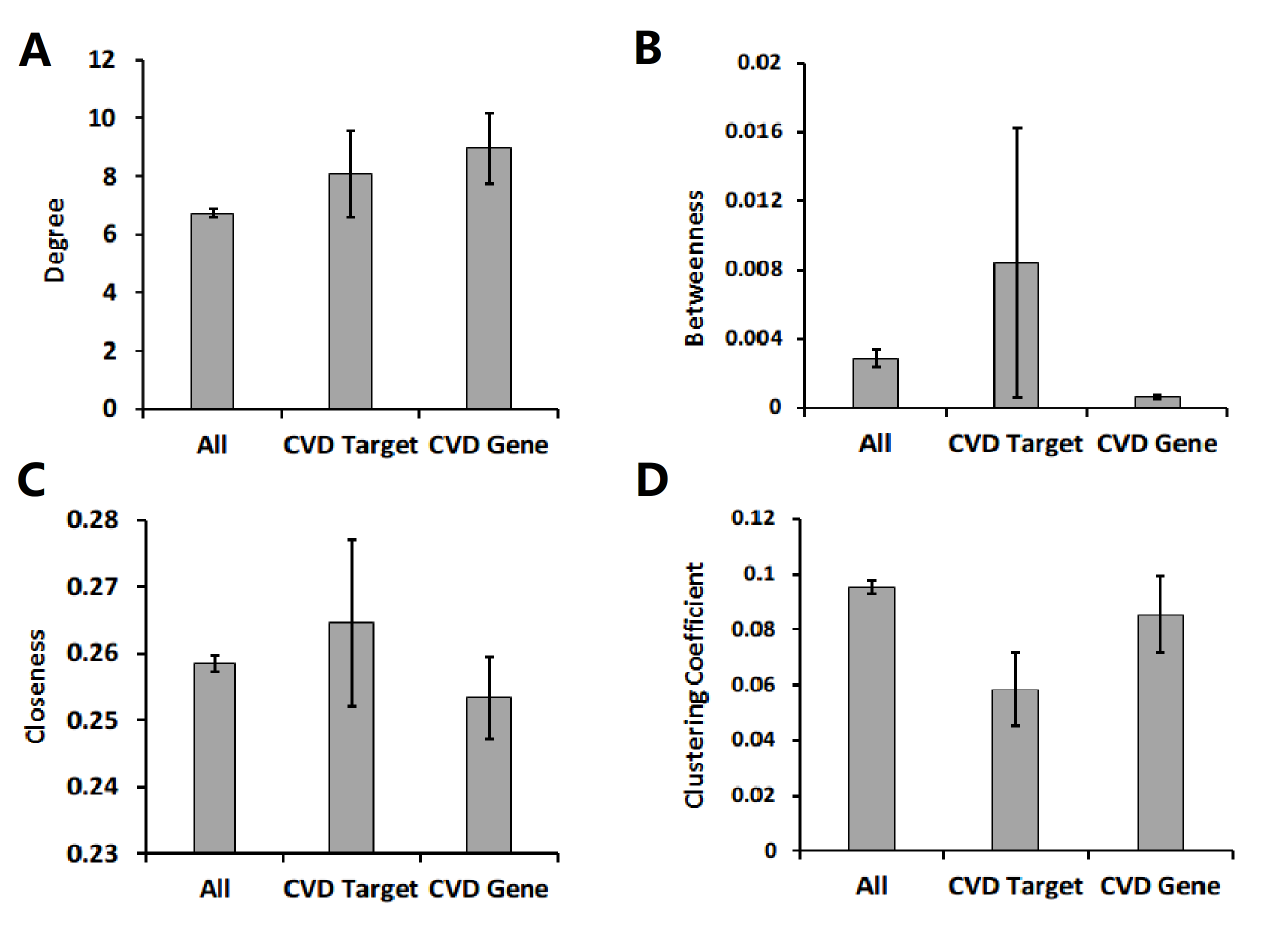


**Supplementary Figure S7.** **Topological features for all proteins, cardiovascular gene products and target proteins in the protein-protein interaction (PPI) network. (a)** Average degree of different classes of proteins in PPI network. **(b)** Average betweenness of different classes of proteins in PPI network. **(c)** Average closeness of different classes of proteins in PPI network. **(d)** Average clustering coefficient of different classes of proteins in PPI network.

**
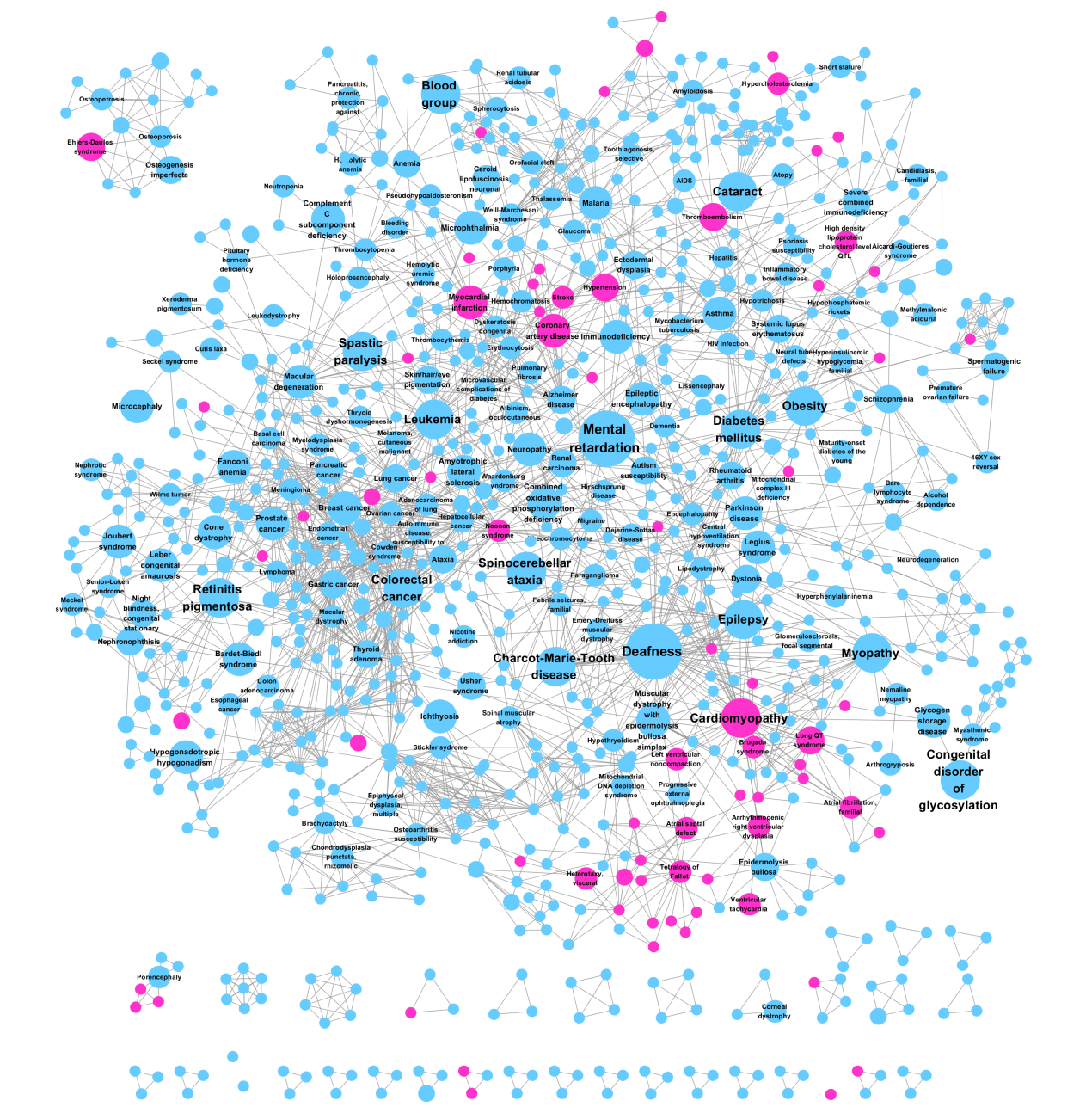
**

**Supplementary Figure S8. Gene disease-disease network (GDD network).** In the GDD network, each node is a disorder, and two disorders are linked if the same gene is involved in both diseases. The size of each node is proportional to the number of genes participating in the corresponding disorder. Pink and green represent cardiovascular disorders and non-cardiovascular disorders.

**
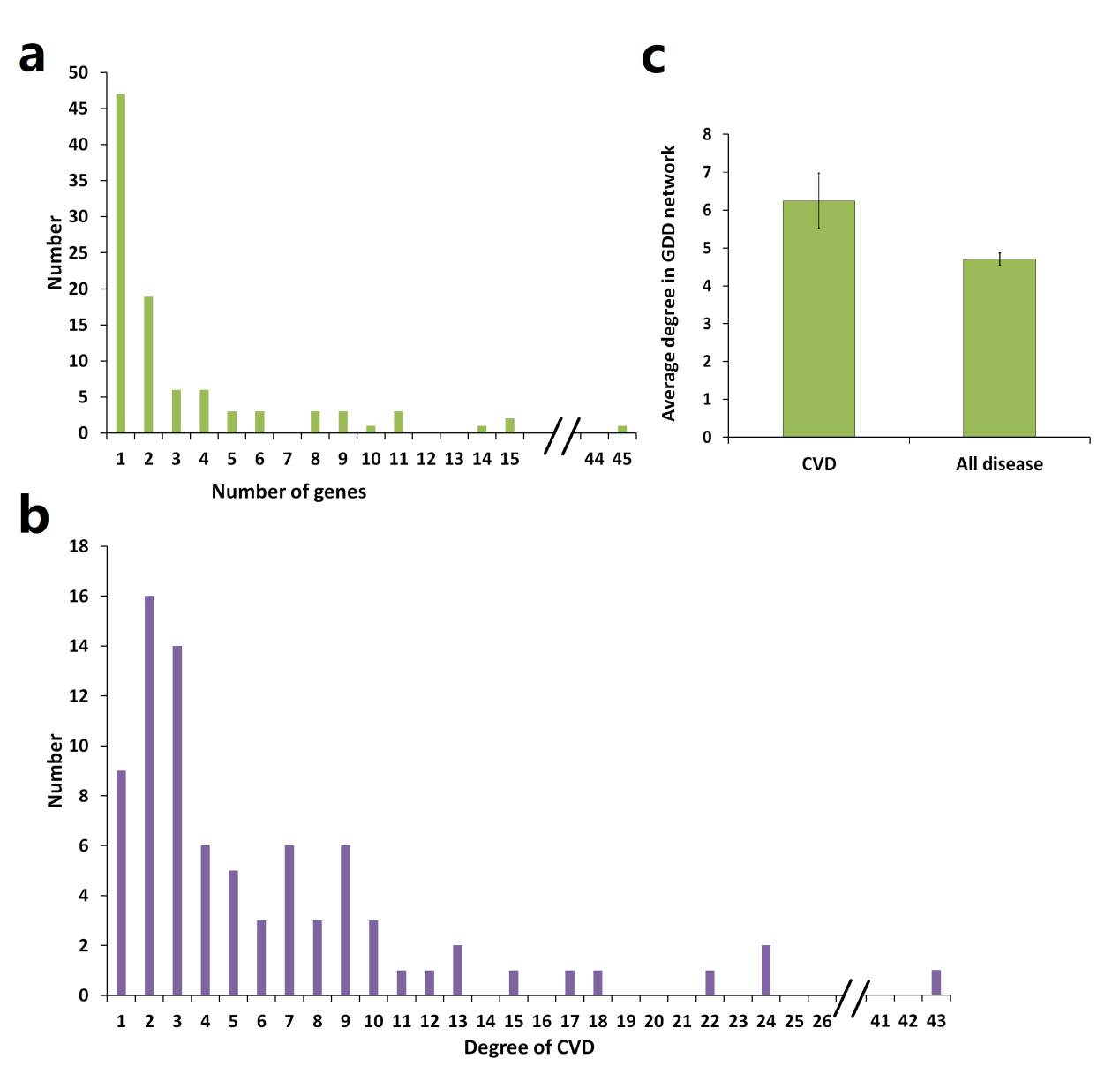
**

**Supplementary Figure S9. Distribution of cardiovascular disorders. (a)** The distribution of number of genes associated with cardiovascular disorders. **(b)** The degree distribution of cardiovascular disorders in the gene disease-disease network (GDD network). (c) The average degree of cardiovascular disorders and all disorders in the GDD network.


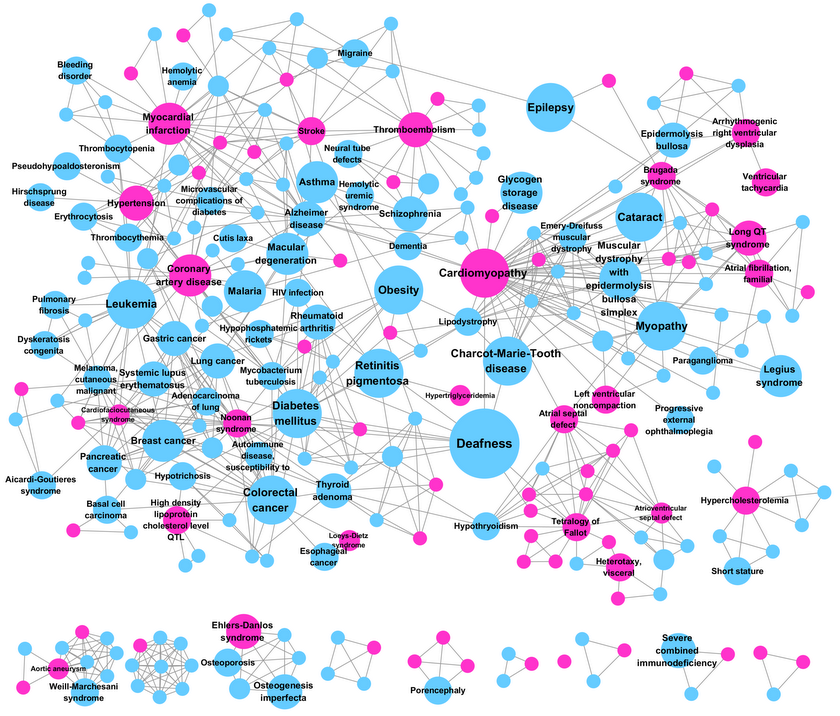


**Supplementary Figure S10. The interaction network of cardiovascular disorders and their direct connected nodes**. Nearly 90% (61 disorders) cardiovascular phenotypes in the biggest component of GGD network are included in this network, indicating the intimate relationships between most CVD. Pink and green represent cardiovascular disorders and non-cardiovascular disorders.


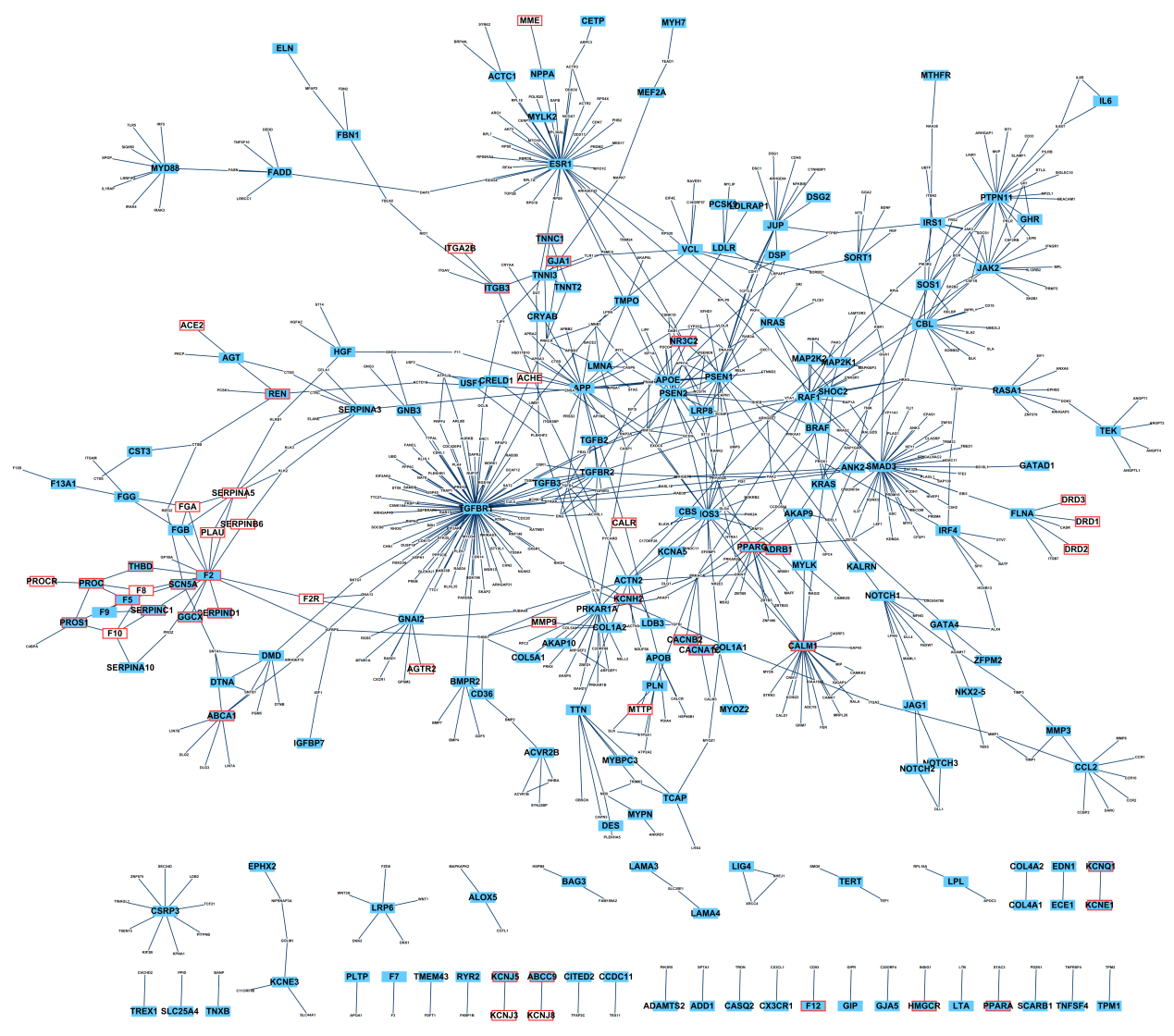


**Supplementary Figure S11. Cardiovascular disease modular network.** The disease modular network is generated by including cardiovascular disease genes and their first-order interactions in the PPI network. The blue-filled rectangles are cardiovascular disease genes. The rectangles with red border are cardiovascular targets. The blue-filled rectangles with red border are both cardiovascular targets and genes. Other proteins are the neighbors of cardiovascular genes. A disease module is composed of the corresponding disease genes and neighbors of these genes. Two examples are provided as Figure 4.


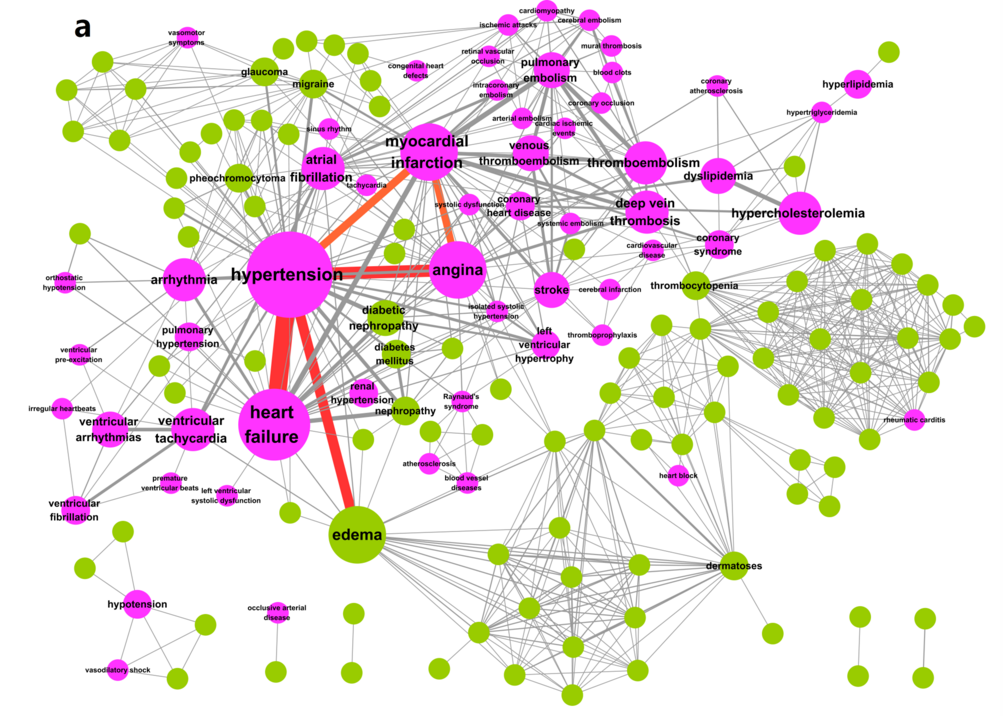


**Supplementary Figure S12.** **Drug disease-disease network (DDD network).** In the DDD network, each node is a disease picked from “indication” field of cardiovascular drug information obtained from the DrugBank database. A link is placed between two disease nodes if there is a drug that treats both. The area of each node is proportional to the number of drugs treating the disease. The thickness of the edge between two nodes is proportional to the number of drugs used for both diseases. Pink and green represent cardiovascular disorders and non-cardiovascular disorders.


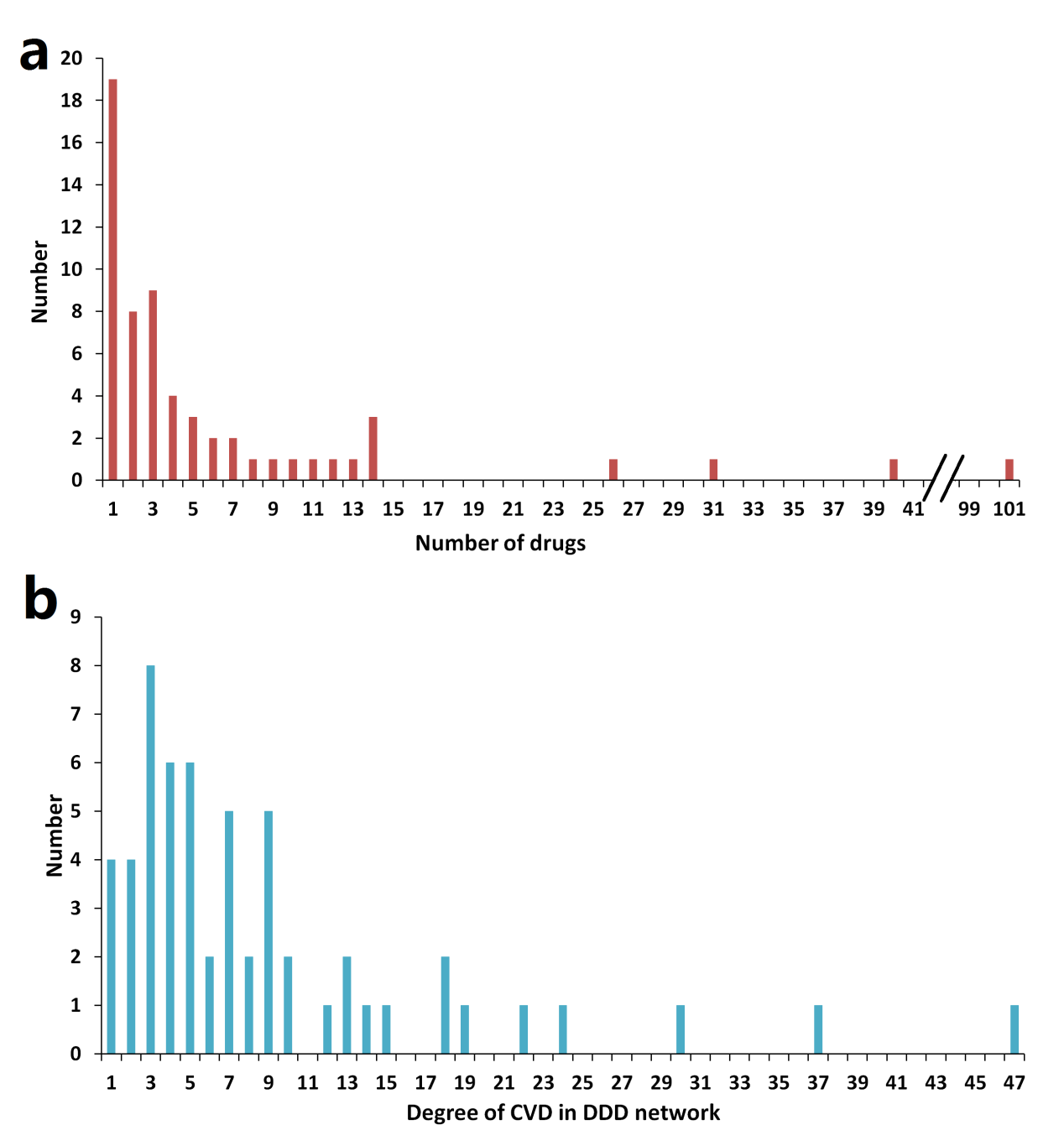


**Supplementary Figure S13. Properties of cardiovascular disorders in the drug disease-disease network (DDD network). (a)** The distribution of drug for cardiovascular disorders. **(b)** The distribution of degrees (related disease number) for cardiovascular disorders.


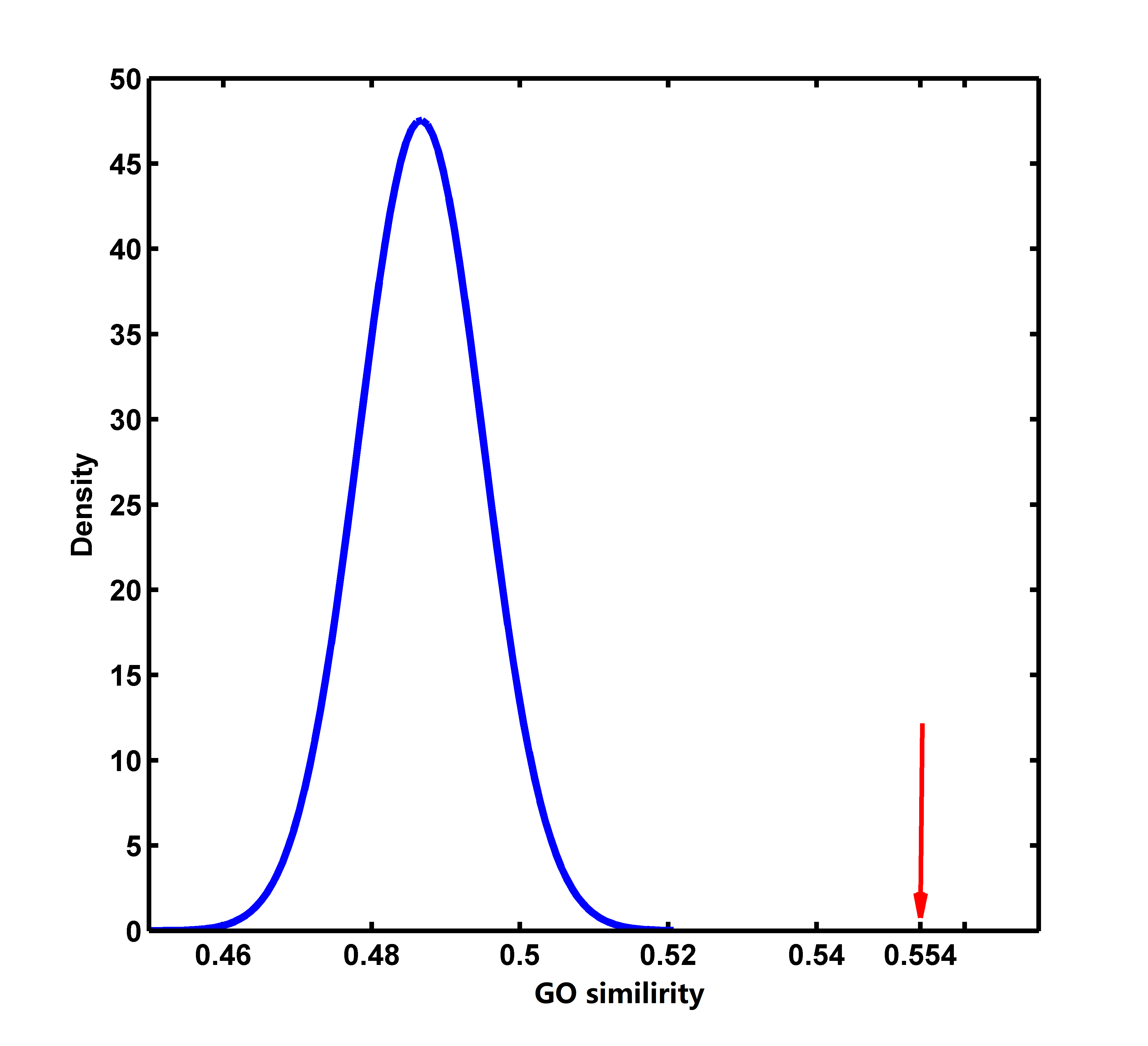


**Supplementary Figure S14.** The co-expression correlation and functional similarity between cardiovascular targets and genes. Red line: observed GO similarity between cardiovascular target and genes. Blue line: GO similarity for random control.

**Supplementary Datasets**

Supplementary Datasets are available at http://sm.nwsuaf.edu.cn/lsp/load_intro.php?site=cvdsp&id=48.

**Supplementary Dataset S1**

Approved cardiovascular drugs and corresponding targets.

**Supplementary Dataset S2**

Experimental cardiovascular drugs and corresponding targets.

**Supplementary Dataset S3**

Extended non-cardiovascular drugs and non-cardiovasuclar targets.

**Supplementary Dataset S4**

Disorder-gene association from OMIM morbid map (January 2013).

**Supplementary Dataset S5**

Cardiovascular disorder-gene association.

**Supplementary Dataset S6**

Cardiovascular target proteins and disorder association.

**Supplementary Dataset S7**

Molecular connections between disease pairs

**Supplementary Dataset S8**

Cardiovascular drug-disease association.

**Supplementary Dataset S9**

Drugs for heart failure and hypertension and their suggested novel drug uses for each other.
